# Resequencing of global *Lotus corniculatus* accessions reveals population distribution and genetic loci, associated with cyanogenic glycosides accumulation and growth traits

**DOI:** 10.1186/s12915-023-01670-7

**Published:** 2023-08-17

**Authors:** Cheng Chen, Kaixuan Zhang, Fu Liu, Xia Wang, Yang Yao, Xiaolei Niu, Yuqi He, Jun Hong, Fang Liu, Qiu Gao, Yi Zhang, Yurong Li, Meijuan Wang, Jizhen Lin, Yu Fan, Kui Ren, Lunhao Shen, Bin Gao, Xue Ren, Weifei Yang, Milen I. Georgiev, Xinquan Zhang, Meiliang Zhou

**Affiliations:** 1 Institute of Crop Sciences, Chinese Academy of Agricultural Sciences, Beijing, 100081 China; 2 National Nanfan Research Institute, Chinese Academy of Agricultural Sciences, Sanya, 572024 China; 3 College of Grassland Science and Technology, Sichuan Agricultural University, Chengdu, 611130 China; 4 Annoroad Gene Technology (Beijing) Co., Ltd., Beijing, 100177 China; 5 Hainan Key Laboratory for Sustainable Utilization of Tropical Bioresource, College of Tropical Crops, Hainan University, Haikou, 570228 China; 6 National Herbage Gempiasm Bank of China, National Animal Husbandry Service, Beijing, 100125 China; 7 Laboratory of Metabolomics, The Stephan Angeloff Institute of Microbiology, Bulgarian Academy of Sciences, Plovdiv, Bulgaria; 8 Center of Plant Systems Biology and Biotechnology, Plovdiv, Bulgaria

**Keywords:** *Lotus corniculatus*, Resequencing, Genomic variation, GWAS, Population structure, Cyanogenic glycosides, Growth traits

## Abstract

**Background:**

*Lotus corniculatus* is a widely distributed perennial legume whose great adaptability to different environments and resistance to barrenness make it an excellent forage and ecological restoration plant. However, its molecular genetics and genomic relationships among populations are yet to be uncovered.

**Result:**

Here we report on a genomic variation map from worldwide 272 *L. corniculatus* accessions by genome resequencing. Our analysis suggests that *L. corniculatus* accessions have high genetic diversity and could be further divided into three subgroups, with the genetic diversity centers were located in Transcaucasia. Several candidate genes and SNP site associated with CNglcs content and growth traits were identified by genome-wide associated study (GWAS). A non-synonymous in *LjMTR* was responsible for the decreased expression of CNglcs synthesis genes and *LjZCD* was verified to positively regulate CNglcs synthesis gene *CYP79D3*. The *LjZCB* and an SNP in *LjZCA* promoter were confirmed to be involved in plant growth.

**Conclusion:**

This study provided a large number of genomic resources and described genetic relationship and population structure among different accessions. Moreover, we attempt to provide insights into the molecular studies and breeding of CNglcs and growth traits in *L. corniculatus*.

**Supplementary Information:**

The online version contains supplementary material available at 10.1186/s12915-023-01670-7.

## Background

Birdsfoot trefoil (*Lotus corniculatus*) is a perennial legume widely distributed and applied around the globe as a kind of high-quality forage legume [1]. The *L. corniculatus* plants exhibit strong stress resistance and can maintain relatively high yield under barren, saline soil, and flooding conditions [2, 3]. Its symbiotic nitrogen fixation with rhizobia makes them an excellent ecological restoration plant, which is widely used in grassland renovation, recovery of vegetation in mined or disturbed areas, and providing understory growth and nitrogen for forests [4, 5]. In addition, its prolonged flowering period makes the use as a honey plant [6]. Biomass and feed quality were the two main screening indicators in the evaluation and selection of forage species [7]. *L. corniculatus* exhibit higher tannin content, which enhanced the nitrogen utilization in livestock, and its feed quality including protein, organic matter, neutral detergent fiber, acid detergent fiber, among others, showed minor difference in *Trifolium pratense* and *Medicago sativa* indicating that it is an excellent forage [8]. From the global cultivated area, the space of *L. corniculatus* appeared considerably lower than that of *M. sativa* and *T. pratense*, which is proportional to its herbage production [2]. Additionally, the reason why *L. corniculatus* is the main cultivated *Lotus* species in the world is that it accumulated more biomass than others species [9]. Therefore, the biomass is particularly vital in *L. corniculatus* breeding. *L. corniculatus* also synthesizes and accumulates cyanogenic glycosides (CNglcs), which are widely present plant secondary metabolites, that can be found in plants of different genera such as *Sorghum bicolor*, *Manihot esculenta*, *Trifolium repens*, *Prunus armeniaca*, and *Eucalyptus robusta* [10, 11]. Therefore, considering the sustainable development of agriculture, the development, utilization, and improvement of germplasm resources of *L. corniculatus* are of great significance.

In plants, CNglcs act as a kind of defense compounds to ensure pathogen and herbivore resistance and nitrogen storage [10, 12]. CNglcs make cyanogenic crops acquire higher resistance to diseases and herbivore; furthermore, CNglcs amygdalin and linamarin are also associated with flavor of food and potential anti-cancer drugs [10, 13–15]. However, the HCN (hydrogen cyanide) released by CNglcs after being hydrolyzed by hydrolase can cause severe toxicity and even death, when the crops with high CNglcs are ingested by humans and domestic animals [16, 17]. The content of CNglcs had been considered in the breeding program of cyanogenic plants like cassava, *T. repens*, and *L. corniculatus* for the health and safety of human and livestock [18–20]. Therefore, it is of particular importance to study the mechanism of the regulation of CNglcs synthesis and their variation range of among different populations in order to use it appropriately, not only for forage breeding but also for the study of other cyanogenic plants. On the other hand, since the biomass of *L. corniculatus* still needs to be improved, we are also highly concerned about its biomass-related growth traits, such as plant height and stem length, which are positively correlated with biomass [19].

In recent decades, the development of sequencing technology enhances our ability to have a clearer understanding of the genetic background of different germplasms, the theoretical basis for their divergence and domestication like rice [21], maize [22], wheat [23], soybean [24], just to name a few. This technique has been used extensively to study plant diversity phenotypes in large natural populations, enhanced our understanding of the relationship between domestication and agronomic traits, to isolate and identify multiple genes controlling essential traits, and speed up the selection process of breeding populations [25–27]. As an allotetraploid and self-incompatible legume, research and utilization of *L. corniculatus* was hindered to some extent [28]. The *Lotus japonicus*, a diploid self-pollination specie, which is closely related to *L. corniculatus* [28–31], possessed a highly efficient genetic transformation system and high-quality reference genome, hence appeared an ideal reference model and auxiliary material for *L. corniculatus*.

In this study, 272 worldwide-collected *L. corniculatus* accessions were sequenced and mapped to *L. japonicus* genome generating 467,831 SNP sites. These SNP sites were used to study the population structure, genetic diversity, selective sweeps, and genetic relationships among *L. corniculatus* accessions. The genes associated with CNglcs synthesis and other growth traits were identified by GWAS and their function was further verified through transient and stable transformation candidate genes into *Arabidopsis* and *L. corniculatus*.

## Results

### Genome-wide variations and population structure of *L. corniculatus*

We collected in total 272 birdsfoot trefoil (*L. corniculatus*) germplasms from 30 different countries, predominantly from eastern Europe and central Asia as well as one *L. frondosus* accession (Table S1). A total of 1.69 Tb raw data as generated, comprising from 11.57 billion reads with an average sequencing depth of 13.08 × coverage and an average 93.82% of mapping rate of the *L. japonicus* genome were retained for SNP calling (Table S1). After data filtering, 467,831 SNPs and 75,962 indels (1–50 bp in length) were identified (Table 1). There are 364,256 SNPs and 73,137 indels in non-coding region, and 103,575 SNPs and 2825 indels in coding sequence (Table 1).

**Table 1:** Genome-wide variations identified in 272 birdsfoot trefoil germplasms

Groups		SNP			Indel	
	Total	Non-coding region	Coding sequence	Total	Non-coding region	Coding sequence
All	467,831	364,256	103,575	75,962	73,137	2825
Group I	403,114	313,982	89,132	69,264	66,663	2601
Group II	467,829	364,255	103,574	75,853	73,032	2821
Group III	466,036	362,898	103,138	75,497	72,693	2804

**Fig. 1:**
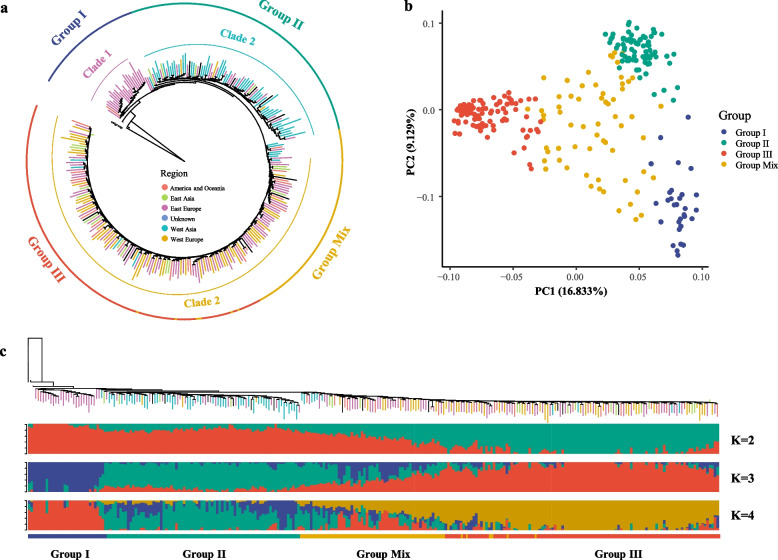
Population structure of resequenced accessions from *L. corniculatus*. **a** Neighbor-joining tree of 273 germplasms, including 272 *L. corniculatus* accessions and 1 *L. frondosus*. **b** PCA of *L. corniculatus* accessions matching the colors shown in (**a**). PC1 and PC2 were first and second components, respectively. **c** ADMIXTURE plot of 272 *L. corniculatus* accessions show three subpopulations (*k* = 2, 3, 4) matches the phylogenetic tree. Colors correspond to the phylogenetic tree grouping

The phylogenetic analysis showed that these 272 birdsfoot trefoil germplasms could be divided into three clades as follows: clade 1, clade 2, and clade 3 (Fig. 1a). As shown in Fig. 1a, clade1 consists mainly of germplasms from East Europe, the germplasms from West Asia are mostly cluster in calde2, and the largest is Clade 3 which covers almost worldwide regions (Fig. 1a, Fig. S1a). By combining phylogenetic analysis, principal component analysis (PCA), and ADMIXTURE analysis, these germplasms are divided into four subgroups: Group I, Group II, Group III, and Group Mix (Fig. 1). Clade1 is the main part of Group I, clade2 is the main part of Group II, and clade 3 contains Group III and Group Mix (Fig. 1a). The PCA analysis can clearly separate Group I, Group II, and Group III in the coordinate with large difference, and Group Mix is scattered among the three (Fig. 1b). The ADMIXTURE analysis of these germplasms showed: Group I, Group II, and Group III mainly come from three different genetic backgrounds, and the genetic background of Group Mix is mixed in different ways when *k* = 3 (Table S2); the genetic background of Group II is partially mixed when *K* = 4, and the other results are similar to *k* = 3 (Fig. 1c).

### Genetic diversity and divergence among *L. corniculatus* subgroups

**Fig. 2:**
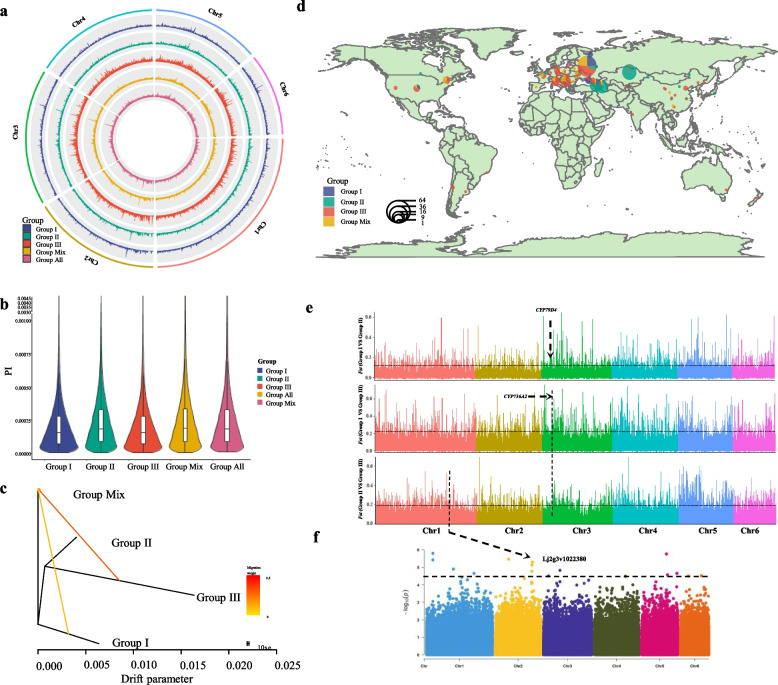
**a** Overview of the nucleotide diversity in all the *L. corniculatus* germplasms and four subgroups: Group I, Group II, Group III, and Group Mix. **b** Violin plot of all the *L. corniculatus* germplasms and four subgroups. **c** The TreeMix population graph of four subgroups: Group I, Group II, Group III, and Group Mix. **d** Geographic distributions of *L. corniculatus* accessions. Each accession is represented by a dot on the map. Each pie chart represents the collection place of *L. corniculatus* accessions, the area size represents the number of accessions. **e** Genome-wide distribution of selective-sweep signals (sliding windows and steps are 10 kb and 5 kb, respectively) identified through comparisons between Group I and Group II (upper panel), Group I and Group III (middle panel), and spring and winter ecotypes (lower panel). The black dashed lines represent the thresholds (top 5% of *F*_*ST*_ values). The *F*_*ST*_ values of the window which harbors *CYP79D4* was 0.138. **f** Manhattan plots of GWAS results (bottom panel) for the plant height trait (September 2020 and May 2021 in LiangShan) on Chr1 and Chr2. The black dashed lines indicate the significance threshold (*p* value = 2.0 × 10^−5^) and black arrow indicates the significant GWAS peak

To further explore the differences between these subgroups, we estimate the nucleotide diversity by calculating PI (the probability that two randomly selected homologous sequences in a population are identical) of the subgroups (Fig. 2a). PI of all these germplasms is 2.38 × 10^−4^, and Group Mix estimates the highest nucleotide diversity (PI = 2.42 × 10^−4^), Group II (PI = 2.37 × 10^−4^), Group III (PI = 2.21 × 10^−4^) and Group I (PI = 1.99 × 10^−4^) decrease successively (Fig. 2b). Then the Treemix analysis found that Group III had strong gene flow to Group Mix and Group II had slightly lighter gene flow to population Group Mix (Fig. 2c). This result is consistent with the analysis result of ADMIXTURE analysis (Fig. 1c), indicating that Group Mix has introgression of Group II and Group III.

From the geographical location of these subgroups, we can see that Group I is mainly distributed in East Europe like Russia, Georgia, Ukraine, Azerbaijan; Group II is mainly harvested from central and western Asian regions Georgia, Azerbaijan, and Kazakhstan; while Group III and Group Mix are mostly distributed in European countries and other continents (Fig. 2d). Four subgroups were distributed in Transcaucasia of Georgia, Azerbaijan, Ukraine, and Russia, indicating that Transcaucasia may be a highly active center for speciation and genetic diversity of *L. corniculatus*. It can be seen from the map that the germplasms of Group II are mainly distributed in the steppe region (Fig. 2c), which has a high nucleotide diversity (PI = 2.37 × 10^−4^), which may be related to the changeable climatic conditions of the steppe and the migration of animals and human nomadic. Group Mix and Group III belong to a same clade in the phylogenetic analysis (Fig. 1a) and are similar in geographical distribution (Fig. 2d). From the analysis result of Treemix and ADMIXTURE analyses (Fig. 2c), we speculate that Group Mix may be a hybrid population that has infiltrated the gene of Group II in Group III. In addition, most commercial cultivars currently available on the market belong to Group III, one cultivar and one landrace are involved in Group II (Table S1). The plant height (Group I = 18.20 ± 0.8414, Group II = 21.39 ± 0.5917, Group III = 20.93 ± 0.4445, Group Mix = 20.31 ± 0.6640; cm) and stem length (Group I = 32.74 ± 1.285, Group II = 37.97 ± 0.8111, Group III = 34.26 ± 0.5157, Group Mix = 35.86 ± 0.8094; cm) and CNglcs (Group I = 44.96 ± 4.273, Group II = 39.41 ± 1.981, Group III = 28.44 ± 1.994, Group Mix = 36.09 ± 3.050; mg/g) of these germplasms matured in July 2019 showed that Group I had the lowest plant height and stem length, while the content of CNglcs was the highest (Fig. S2a, b). Group II showed highest plant height and stem length, and the content of CNglcs appeared average (Fig. S2a, b, c). Group III accumulated moderate biomass, but has the lowest CNglcs content (Fig. S2a, b, c). Since the worldwide distribution of *L. corniculatus* is mainly related to human activities [5], low CNglcs are more cater to breeders’ preferences, so we suggest that the content of CNglcs is related to Group III distribution in the world.

To further investigate the divergences among Group I, Group II, and Group III, the selective sweeps were scanned using population fixation statistics (*F*_*ST*_ values with 10-kb sliding windows, a step of 5 kb). In total, 1856 regions with the top 5% of *F*_*ST*_ values were considered as candidate divergence sweeps between Group I and Group II, Group I and Group III, and Group II and Group III (Fig. 2e and Table S3-5). The selective-sweep regions between Group I and Group II, Group I and Group III, and Group II and Group III, with 1733, 2185, 2338 genes, respectively. Gene annotation and GO annotation were performed, and we found that these selective-sweep regions were mainly involved in biological process such as metabolic process and cellular process, molecular function like binding and catalytic activity (Table S6-8, Fig. S2d). The genes located in these selective-sweep regions could potentially play important roles. CNglcs synthesis gene *CYP79D4* [32] located in divergence sweeps Chr3: 8565001–8575000 (*F*_*ST*_ = 0.138795) and Chr3:8570001–8580000 (*F*_*ST*_ = 0.133886) between Group I and Group II (Fig. 2e). Another CNglcs synthesis gene *CYP736A2* [32] located in divergence sweep Chr3: 8825001–8830000 was found both in divergence sweeps between Group I and Group III (*F*_*ST*_ = 0.401206) and Group II and Group III (*F*_*ST*_ = 0.214295). These selective-sweep regions may be one of the reasons for the differences in CNglcs content in these subgroups. We further performed GWAS analysis for 241 germplasms of Group II and Group III (Table S9), by using MLMM, Blink, and FarmCPU method to detect locus related to 3 traits about CNglcs content and growth. A selective-sweep for Group II and Group III Chr2: 34685001–34690000 (*F*_*ST*_ = 0.279585) associated with plant height (May 2021 in LiangShan) located chromosome 2, harboring a genes *Lj2g3v1022380* (Chr2_ 34708630, -log10 *P* = 5.12) (Fig. 2e, Fig. S12 and Table S12). Its homolog *AT1G65690* was involved in ABA signaling and biosynthesis, suggesting that this site could related to SL [33].Those results indicated that the divergences among these three groups are related to CNglcs content and biomass trait.

### Genome-wide association with CNglcs

**Fig. 3:**
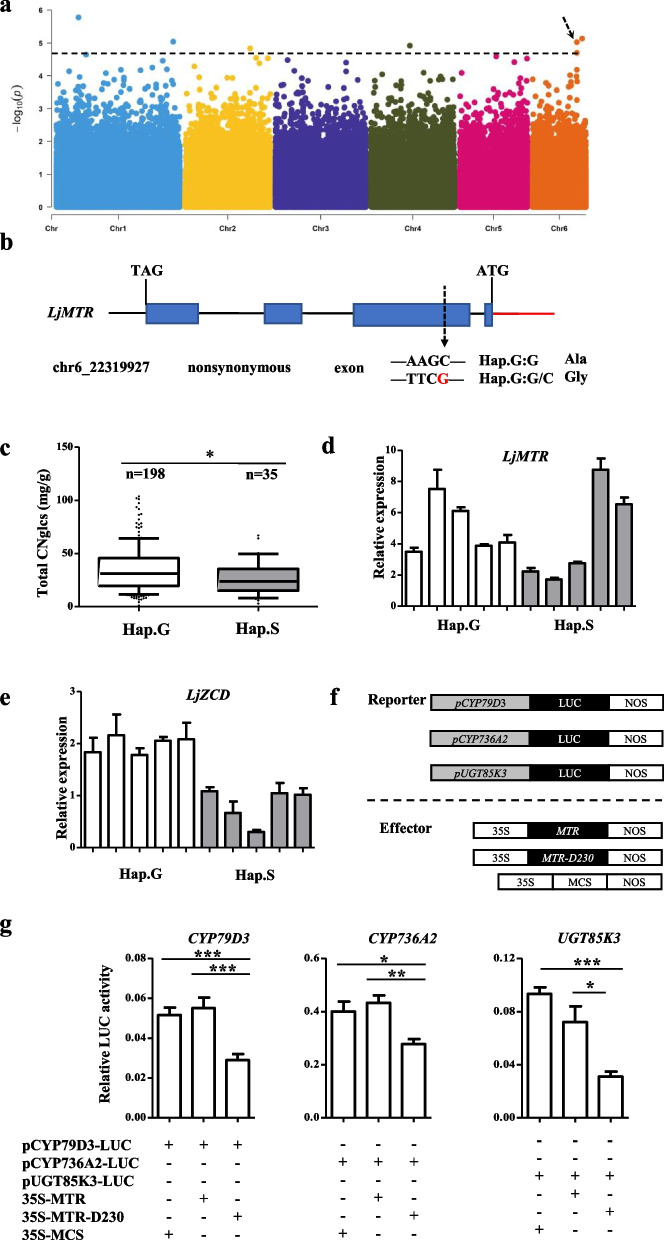
GWAS identification of candidate genes associate with CNglcs content. **a** Manhattan plots for total CNglcs content in 241 accessions using MLMM. The black dashed lines indicate the significance threshold (*p* value = 2.0 × 10.^−5^) and black arrow indicates the significant GWAS peak. **b** Gene model and SNPs of LjMTR. Exons, introns and promote are represented by blue boxes, black lines and red line, respectively. The non-synonymous SNP is marked by a black arrow. Hap.G, haplotype G (GG); Hap.S, haplotype S (GC). **c** Box plot of total CNglcs content, the white box and gray box represent Hap.G (GG) and Hap.S (GC), respectively. The significance of difference was derived with two-tailed t-test (*P < 0.05). **d–e** Relative expression of *LjMTR* and *LjZCD* in different accessions carrying Hap.G and Hap.S, respectively. The white bar and gray bar represent Hap.G and Hap.S, respectively. **f** Schematic diagram of reporter and effector in *Arabidopsis* protoplast transactivation assays. Reporter is the fusion of the *CYP79D3*, *CYP736A2*, and *UGT85K3* promoter with firefly luciferase gene (LUC). The *LjMTR*, site mutated *LjMTR-D230* and *LjZCD* fused with the CaMV 35S promoter respectively to from effectors. NOS, the transcriptional terminator of the nopaline synthase gene from Agrobacterium tumefaciens. **g** Luciferase assays of *LjMTR*, and site mutated *LjMTR-D230* regulate the expression of *CYP79D3*, *CYP736A2*, and *UGT85K3*. The empty vector was used for control. Values represent the mean ± SE of triplicate experiments. Significant differences between values are indicated with different letters (*, *P* < 0.05; **, *P* < 0.005; ***, *P* < 0.0001 one-way ANOVA)

*L. corniculatus* contains two CNglcs, lotaustralin and linamarin [34, 35]. We performed GWAS for the relative content of total cyanogenic glycosides, lotaustralin and linamarin in Group II and Group III (Table S9 and Fig. S3-5). One significant association with the total CNglcs content was identified on chromosome 6, Chr6_22318549 (-log10 *P* = 5.02) (Fig. 3a). The candidate region from 22.28 Mb to 22.34 Mb (60 kb) contains 17 candidate genes (Table S10). The peak SNP (Chr6_22318549, T/C) is located at the intron of *LjMTR* (*Lj6g3v1948640*). By analyzing all SNP sites on *LjMTR*, we found a non-synonymous mutation site chr6_22319927 (G/C) in the exon of *LjMTR* caused an amino acid change from Ala to Gly (Fig. 3b). These accessions can be divided into two major haplotypes Hap.G (GG) and Hap.S (GC), and the content of CNglcs in Hap.G is significantly higher than that in Hap.S (Fig. 3c). The expression of CNglcs synthesis genes were also higher in Hap.G than in Hap.S (Fig. S6a). Indicating that this SNP site may be related to the content of CNglcs. Additionally, we detected the expression level of all the candidate genes in Hap.G and Hap.S plants to detect if there are differentially expressed genes in these two haplotypes (Fig. S6b). There was no significant difference in the expression of *LjMTR* suggesting that the difference in CNglcs content might be due to the change in protein structure caused by non-synonymous mutation (Fig. 3d). The higher expression of *LjZCD* (*Lj6g3v1946530*) in Hap.G than in Hap.S (Fig. 3e) indicates that this gene may be involved in the regulation of CNglcs synthesis.

The *LjMTR* homolog of *AtOSCA3*, an early-responsive to dehydration gene, which is involved in plant growth and response to drought, salt and pathogen stress [36–39]. Here, we performed transient transcriptional activation assay to detect whether this site mutation would have an effect on the transcription of CNglcs key synthase genes by Dual-Luciferase Reporter system (Fig. 3f). The results showed that unmutated *LjMTR* had no significant effect on the expression of *CYP79D3*, *CYP736A2*, and *UGT85K3*, but the mutated *LjMTR-D230* could significantly reduce the expression of CNglcs synthesis genes (Fig. 3g). Hence, the non-synonymous at chr6: 22319927 (G/C) is responsible for the reduction of CNglcs content.

**Fig. 4:**
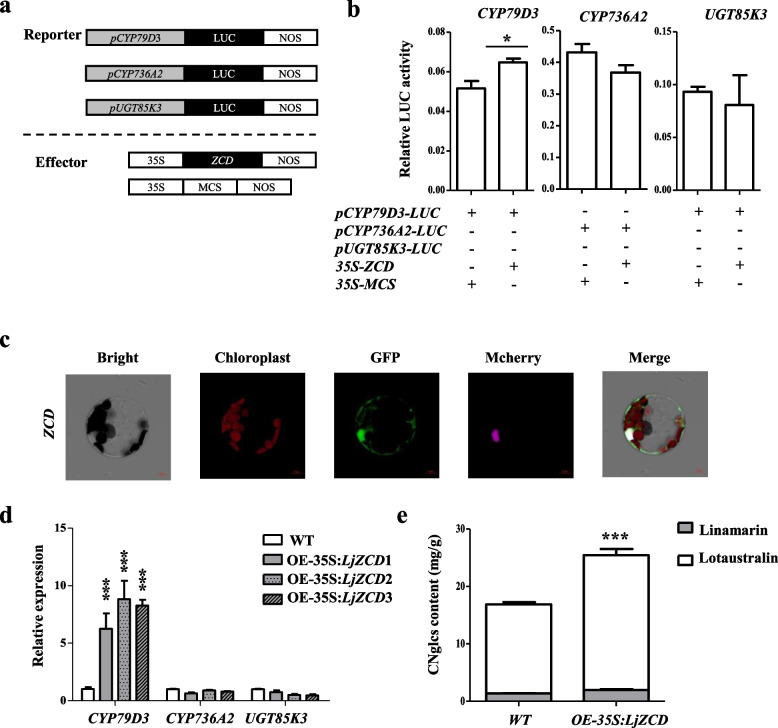
Function identification of *LjZCD* on CNglcs content*. ***a** Schematic diagram of reporter and effector in *Arabidopsis* protoplast transactivation assays. Reporter is the fusion of the *CYP79D3*, *CYP736A2*, and *UGT85K3* promoter with firefly luciferase gene (LUC). The *LjZCD* fused with the CaMV 35S promoter respectively to from effectors. NOS, the transcriptional terminator of the nopaline synthase gene from Agrobacterium tumefaciens. **b** Luciferase assays of *LjZCD* regulate the expression of *CYP79D3*, *CYP736A2*, and *UGT85K3*. The empty vector was used for control. Values represent the mean ± SE of triplicate experiments. Significant differences between values are indicated with different letters (*, *P* < 0.05; **, *P* < 0.005; ***, *P* < 0.0001 one-way ANOVA). **c** Subcellular localization of LjZCD protein in *Arabidopsis*. LjZCD-GFP constructs were co-transformed with AtH2B-Mcherry into *Arabidopsis* mesophyll protoplasts and examined by confocal laser scanning microscopy. Confocal micrographs are shown brightfield, chloroplast auto-fluorescence, green fluorescent protein (GFP), and the merged images from left to right. AtH2B-Mcherry was used as nuclear localization maker. Bar, 5 μm. **d** The relative expression of CNglcs genes in wild type (*WT*) and 3 lines of *OE-35S*::*LjZCD*. **e** The contents of two cyanogenic glycosides in *WT* and 3 lines of overexpression material *OE-35S::LjZCD*. The white box and gray box indicate Lotaustralin and Linamarin, respectively

Another candidate gene *LjZCD* was homolog of *AtBRCA1* (breast cancer susceptibility gene 1), which represses RRTF1 transcription factor and ROS-responsible genes under drought stress [40]. The dual-luciferase reporter system was used for transient transcriptional activation assay to check the influence of *LjZCD* on CNglcs synthesis. The results showed that *LjZCD* up-regulated the expression of CYP79D3 and had no significant effect on *CYP736A2* and *UGT85K3* (Fig. 4a, b). Subcellular localization showed that *LjZCD* protein was located in both nucleus and cytoplasm (Fig. 4c). Furthermore, we overexpressed *LjZCD* in *L. japonicus* (Fig. S7A, B), the overexpressed material showed a higher CNglcs content and relative expression of *CYP79D3* (Fig. 4D, E). Therefore, we propose that *LjZCD* may be involved in the regulation CNglcs content by upgrading the CNglcs synthetic gene *CYP79D3*.

### Genome-wide association with stem length

We recorded the growth traits stem length (SL) of 241 germplasms (Group II & Group III) in two farms over 2 years period (2019–2020 in Beijing, 2020–2021 in Liangshan) (Table S11), then GAWS analysis was carried out for these growth traits using MLMM, Blink and FarmCPU method (Fig. S8-12).

**Fig. 5:**
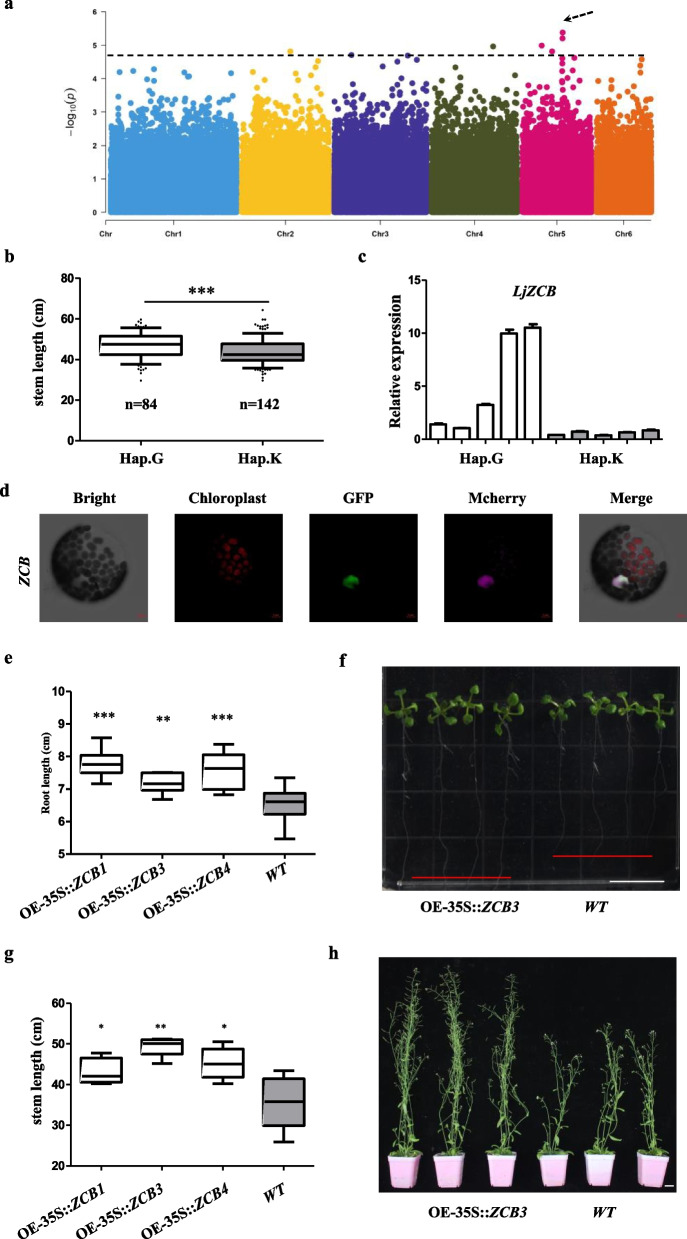
GWAS identification of candidate genes associate with stem length. **a** Manhattan plots for stem length in 241 accessions using MLMM. The black dashed lines indicate the significance threshold (*p* value = 2.0 × 10^–5^) and black arrow indicates the significant GWAS peak. **b** Box plot of stem length, the white box and gray box represent Hap.G and Hap.K, respectively. **c** Relative expression of *LjZCB* in different accessions carrying Hap.G and Hap.K, respectively. The white bar and gray bar represent Hap.G and Hap.K, respectively. **d** Subcellular localization of LjZCB protein in *Arabidopsis*. LjZCB-GFP constructs were co-transformed with AtH2B-Mcherry into *Arabidopsis* mesophyll protoplasts and examined by confocal laser scanning microscopy. Confocal micrographs are shown brightfield, chloroplast auto-fluorescence, green fluorescent protein (GFP), and the merged images from left to right. AtH2B-Mcherry was used as nuclear localization maker. Bar, 5 μm.** e** The box plot of *Arabidopsis* root length in *WT* e and 3 lines of *OE-35S*::*LjZCB* 7 days after germination.** f** The box plot of *Arabidopsis* stem length in *WT* and 3 lines of *OE-35S*::*LjZCB* 50 days after germination.** g** Image of root length of *WT* and *OE-35S::LjZCB* line 3 *Arabidopsis* 7 days after germination. White line represent bar, 2 cm. **h** Image of stem length of *WT* and *OE-35S*::*LjZCB* line 3 *Arabidopsis* 50 days after germination. White line represent bar, 2 cm. The significance of difference was derived with two-tailed *t*-test (*P < 0.05, **P < 0.01, and ***P < 0.001)

Of all the results in SL, multiple significant SNPs were found at chromosome 5, which was related to SL in May 2020 BeiJing (SL_May.20B) (Fig. 5A). The region (chr:5 19.86–19.92 Mb) 30 kb upstream and downstream of peak (chr5_19895618, -log10 *P* = 5.37) harbors 7 candidate genes (Table S11). We analyzed SNP locus in this region and found an SNP site chr5_19909005(G/T) located at exon of *Lj5g3v1222610* causing a synonymous mutation. According to this site, we can divide the materials into two haplotypes Hap.G (GG) and Hap.K (GT), and the SL of Hap.T is significantly higher than that of Hap.K (Fig. 5b). The expression level of *Lj5g3v1222610* in these 7 genes was positively correlated with SL in the two haplotypes (Fig. 5c and Fig. S13a), indicating this gene may promote stem length.

The *LjZCB* (*Lj5g3v1222610*) encode a RING/FYVE/PHD zinc finger protein, and its homolog *AT1G33420* and *AT1G66170* are required for male meiosis [41, 42]. And RING/FYVE/PHD zinc finger protein not only participates in male meiotic chromosome condensation but also can regulate expression of CAP-D3, a condensin gene that mediate vegetative growth and fertility defects in *Arabidopsis* [43]. The subcellular localization of *LjZCB* showed that it was localized in the nucleus which is consistent with other RING/FYVE/PHD zinc finger proteins (Fig. 5d). Three transgenic lines (*OE-35S::ZCB1*, *OE-35S::ZCB3*, *OE-35S::ZCB4*) were obtained by overexpression of *LjZCB* in *Arabidopsis* (Fig. S7c, d); the transgenic lines showed a longer root length than the wild type at seedling stage and longer stem length (Fig. 5e–h). Hence, it could be proposed that *LjZCB* was involved in the regulation of SL.

### Genome-wide association with plant height

We employed GWAS analysis by MLMM, Blink, and FarmCPU method for 241 accessions to identify potential gene locus that are prominently correlated with the growth traits plant height (PH) in two farms over 2 years (2019–2020 in Beijing, 2020–2021 in Liangshan) (Table S9 and Fig. S14-18).

**Fig. 6:**
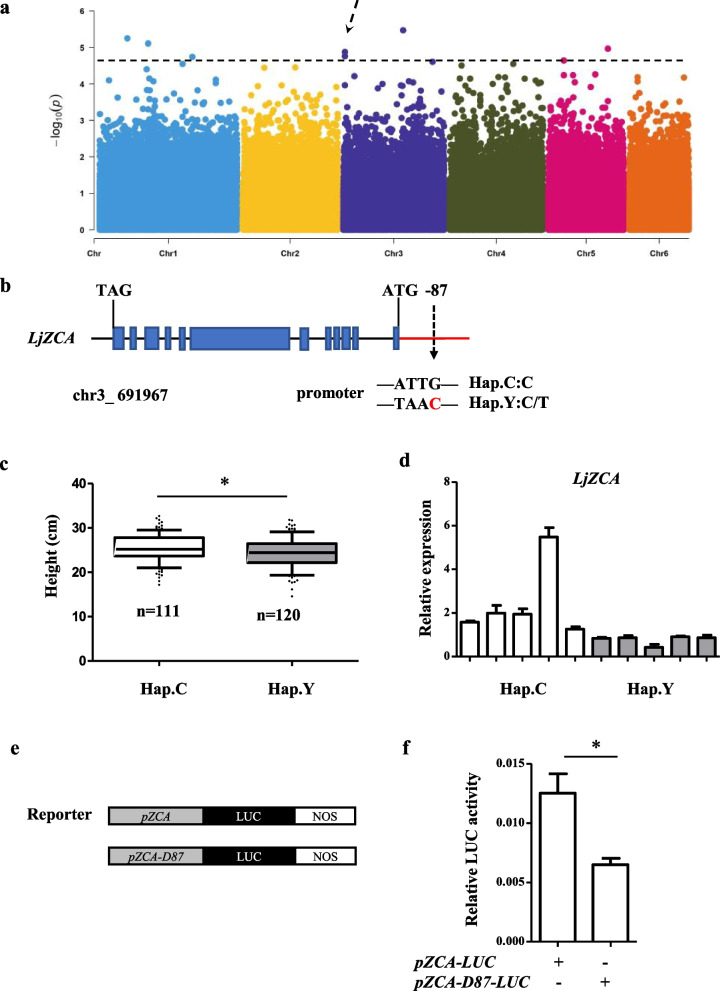
GWAS identification of candidate genes associate with plant height. **a** Manhattan plots for plant height in 241 accessions using MLMM. The black dashed lines indicate the significance threshold (*p* value = 2.0 × 10.^−5^) and black arrow indicates the significant GWAS peak. **b** Gene model of *Lj3g3v0075610*. Exons, introns, and promoter are represented by blue boxes, black lines, and red line, respectively. The SNP in promoter is marked by a black arrow. **c** Box plot of stem length, the white box and gray box represent Hap.C and Hap.Y, respectively. The significance of difference was derived with two-tailed *t*-test (*P < 0.05, **P < 0.01, ***P < 0.001). **d** Relative expression of *Lj3g3v0075610* in different accessions carrying Hap.C and Hap.Y, respectively. The white bar and gray bar represent Hap.C and Hap.Y, respectively. **e** Schematic diagram of reporter and effector in *Arabidopsis* protoplast transactivation assays. The *LjZCA* promoter (*pZCA*) and mutated *pZCA-D87* fused with firefly luciferase gene (LUC), respectively. NOS, the transcriptional terminator of the nopaline synthase gene from Agrobacterium tumefaciens. **f** Luciferase assays of *pZCA*, and *pZCA-D87*. Values represent the mean ± SE of triplicate experiments. Significant differences between values are indicated with different letters (*, *P* < 0.05 one-way ANOVA)

The PH in September 2019 BeiJing (PH_Sep.19B) also identified a signal region chromosome 3: 0.67–0.73 Mb (the peak SNP chr3_706642, -log10 *P* = 4.88) (Fig. 6a), harbors 7 candidate genes (Table S13). An SNP mutation site chr3_ 691967 (C/T) located at 87 bp upstream of *Lj3g3v0075610* (*LjZCA*, disabled the CAAT box (Fig. 6b). The Hap.C harbors an intact CAAT box in promoter showed a longer stem length, while Hap.Y heterozygous with the absence of CAAT box was shorter (Fig. 6C). We further preformed qPCR in Hap.C (CC) and Hap.Y (CT) and identified that the expression of *LjZCA* differs significantly in two haplotypes suggesting that this SNP mutation site might regulate plant height by affecting downstream gene expression (Fig. 6d and Fig. S13B).

The SNP mutation site chr3_ 691967 (C/T) damaged the CAAT box, a cis-acting element and enhancer region, which can influence the frequency of transcriptional initiation [44]. The downstream gene *LjZCA* encodes a SET-domain protein, a class of proteins involved in epigenetic control of gene expression and act as histone methyltransferases. *AtSUVH1*, a homolog of *LjZCA*, was verified to positively regulate plant growth and development [45]. We cloned the 1000 bp upstream promoter sequence of *LjZCA* and performed site-directed mutation at the 87 bp upstream of *Lj3g3v0075610* and inserted the two fragments into the LUC expression vector LUC0800. The results of transient transcriptional activation showed that the vector with complete CAAT box promoter showed significantly higher expression activity (Fig. 6E, F). Thus Hap.C showed a higher relative expression of *LjZCA*, suggesting that this SNP mutation of CAAT box caused the decrease of *LjZCA* expression and reduced the PH.

Another GWAS site (chr3_11234226, -log10 *P* = 5.93) in September LiangShan (PH_Sep.20L) was also identified (Fig. S17). On its candidate region, chr:3 11.20–11.26 Mb harbors *Lj3g3v0937760* (Table S14), a CTP synthase which plays an important role in plant development [46], suggesting that this site may also relate to PH.

## Discussion

With the development of gene sequencing techniques and bioinformatics tools in recent decades, a variety of molecular marker-assisted selective breeding methods have been derived, which considerably improves the efficiency of breeding. The *L. corniculatus* is a widely distributed and utilized leguminous plant; however, the genetic background of its germplasm resources and the genetic relationship between each other are not clear. This greatly restricts the process of human selection and utilization. In our study, 272 *L. corniculatus* germplasms worldwide were resequenced, and 467,831 SNPs and 75,962 indels were obtained by mapping *L. japonicus* reference genome.

According to the SNP sites obtained by resequencing, phylogenetic, PCA, and structure analysis suggest that these germplasms could be divided into four groups, Group I, Group II, Group III, and Group Mix (Fig. 1). The germplasms of these three subgroups were distributed in Transcaucasia (Fig. 2a), making it a center of genetic diversity of *L. corniculatus*. Studies have shown that Transcaucasia is one of the origin centers of alfalfa and chickpea [47, 48]. However, we need more abundant germplasm resources and more accurate genetic information to further study whether Transcaucasia is the origin of *L. corniculatus*. PI of all these germplasms is 2.38 × 10^−4^ and the linkage disequilibrium (LD) decay distance (*r*^2^ = 0.1) of *L. corniculatus* was about 1 kb (Fig. 2a, b; Fig. S1c), which has lower nucleotide diversity than autotetraploid alfalfa, and diploid soybean and chickpea [24, 47–50]. The nucleotide diversity of *L. corniculatus* is more abundant, which may be related to its allotetraploid and self-incompatible [28]. There were significant differences in geographical distribution and agronomic traits among Group I, Group II, and Group III (Fig. 2d; Fig. S2a, b, c). The selective seeps screened by *Fst* values include genes associated with CNglcs synthesis and plant growth and development (Fig. 2b, c), all of which are related to human preferences. Throughout history, humans have tended to breed plants that fit their preferences and hence to spread them around the world when they moved. The Group III accessions accumulated moderate biomass amounts, but their lowest cyanogenic glycoside content make it more wildly distributed than two other groups. Since the transmission of *L. corniculatus* is closely related to human activities [5], we infer that the distribution of different *L. corniculatus* germplasms is related to human selection.

The genes for biosynthesis of CNglcs have been identified in *L. japonicus*, *S. bicolor*, *M. esculenta*, and *P. amygdalus* among others [32, 51–55]. A completion of the almond reference genome reveals the domestication of bitter to sweet kernel trait by the decrease of CNglcs amygdalin and identified a bHLH protein involved in the regulation of CNglcs synthesis gene [14]. We isolated a high CNglcs haplotype (Hap.G) and a low CNglcs (Hap.S) in a non-synonymous mutation by GWAS (Fig. 3a–d). Transient transcriptional activation experiments were performed to verify that this SNP mutation would cause changes in *LjMTR* protein and reduce the expression level of CNglcs synthesis genes (Fig. 3f, g). Another differentially expressed gene *LjZCD* in the two haplotypes showed positive regulation of *CYP79D3* through transient transcriptional activation and over expression in *L. japonicus* (Fig. 4). Both the homolog of these two genes *AtOSCA3* and *AtBRCA1* were drought response genes [36, 40], and it has been reported that drought stress can induce the synthesis of CNglcs [56], suggesting that these two proteins may regulate the synthesis of CNglcs in response to drought. There are few studies on the regulation of CNglcs synthesis, and this study enriches related studies on CNglcs synthesis.

The growth traits plant height (PH) and stem length (SL) were complex traits controlled by multiple genes. We isolated and identified several of SNPs using two years of field data from two cultivation places (Fig. S8-12, 14–18). And we identified *LjZCB* and mutation site (chr3_ 691967 (C/T) damaged the CAAT box) from these candidate genes and SNPs (Figs. 5, 6). No overlapping GWAS sites were found in multi-point field data over 2 years, possibly because *L. corniculatus* has strong heterozygosity and genetic diversity. Its phenotype is considerably affected by the environment, which also shows that heterozygosity may be more conducive to plant adaptation to the environment.

## Conclusion

**Fig. 7:**
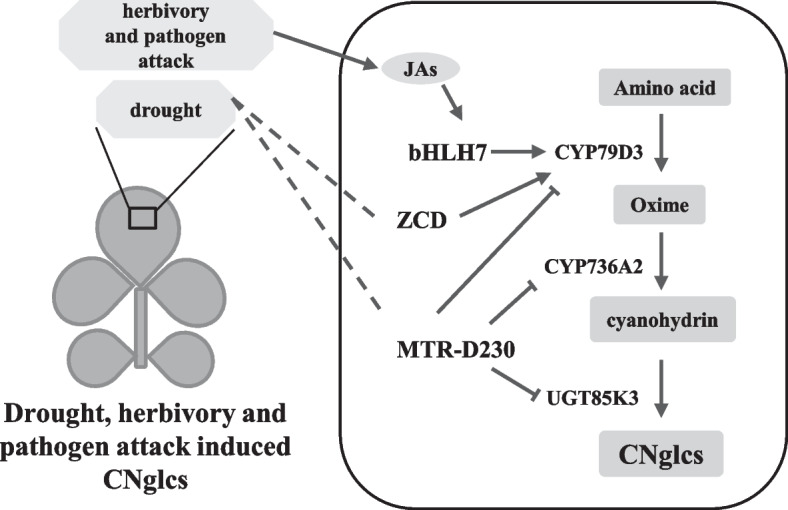
A proposed model of drought, herbivory, and pathogen attack modulates CNglcs. When herbivory and pathogen attack leaves, jasmonic acids (JAs) are induced to activate the expression of the transcription factor bHLH7 which make the CNglcs synthase gene *CYP79D3* highly expressed, thus increasing the content of CNglcs. ZCD increased the expression of *CYP79D3*, and the mutant protein MTR-D230 was able to impress the CNglcs synthase genes *CYP79D3*, *CYP736A2*, and *UGT85K3*. Drought may increase the CNglcs content by affecting the expression of ZCD and MTR. Solid lines indicate that it has been verified and dashed lines indicate that it has not been verified

In summary, our study provides large genomic resource and attempted to reach the point of a better understanding of population structure, geographic distribution, divergence, genetic diversity centers, and factors controlling the spread of *L. corniculatus*. Various factors affect the synthesis of CNglcs [10]. Only the pathway of transcription factor bHLH7 mediated by jasmonate has been explicitly analyzed for the regulation of CNglcs [57], and there has little work on the regulation of CNglcs by non-biogenic factors. Through genome-wide association analysis, we identified a mutant protein MTR-D230 that reduces CNglcs synthesis and a zinc finger protein ZCD that promotes CNglcs synthesis and speculated that this may be related to the regulation of CNglcs by drought (Fig. 7). In addition, we also identified growth-related genes *ZCA* and *ZCB*. This work provides a theoretical basis for future breeding programs for *L. corniculatus*.

## Methods

### Plant materials and phenotyping

A total of 272 birdsfoot trefoil (*L. corniculatus*) and one *Lotus frondosus (Freyn) Kupr.* accessions were obtained from National Herbage Gempiasm Bank of China (Beijing, China). The 272 *L. corniculatus* accessions were planted in Beijing (Beijing, 40° 23′ N, 116° 29′ E), which belongs to temperate monsoon climate. And 241 accessions in Liangshan (Sichuan province, 27° 59′ N, 102° 50′ E), which belongs to subtropical plateau monsoon climate. Each individual was planted in 60 cm square, 6 individual plants for each accession, and the spacing between different lines is 40 cm. For phenotyping experiments in Beijing, we measured 3 times, in July 2019, September 2019, and May 2020, and 2 times in Liangshan, in September 2020 and May 2021, respectively. The phenotypes including the plant height and stem length were measured in 3 individual plants for each accession in full-bloom stage.

### DNA extraction and sequencing

The *L. corniculatus* seedings were used for genomic DNA extraction by the cetyltrimethylammonium bromide (CTAB) method [58]. Sequencing library was constructed by 2 μg genomic DNA for each accession. For library construction, 10 × FD, Fragmentase and other reagents were added to qualified DNA samples using Annoroad® Universal DNA Fragmentase kit V2.0 (AN200101-L); Annoroad® Universal DNA Library Prep Kit V2.0 (AN200101-L) was used for end repair and joint addition; then different proportions of magnetic beads were used to select corresponding DNA fragments, and the target fragments were enriched by PCR. After library construction, Qubit 3.0 was used for preliminary quantification, and the library was diluted to 1 ng/μl Agilent 2100 was then used to detect the insert size of the library. After the insert size met the expectations, Bio-rad CFX 96 fluorescence quantitative PCR, Bio-RAD KIT iQ SYBR GRN assay was used and q-PCR was performed to accurately quantify the effective concentration of the library (effective concentration of the library > 10 nM) to ensure Library quality. Cluster and sequencing were performed on NovaSeq 6000 S4 platform using NovaSeq 6000 S4 Reagent Kit V1.5. Double-ended sequencing (PE) was run to obtain 150 bp double-ended sequencing reads. Raw data of fastq format were filtered to obtain high-quality Clean Reads by SOAPnuke-1.5.6.

### Reads alignment and variant calling

The sequencing reads for each accession were mapped to the *L. japonicus* MG20 reference genome version 3.0 [59] (http://www.kazusa.or.jp/lotus/index.html) using BWA mem Version: 0.7.15-r1140 with default parameters. The mapping results were sorted and filtered the low quality (MQ < 30) reads to get SNPs and small indels by Samtools Version: 1.3.1. UnifiedGenotyper module of GenomeAnalysisTK-3.7.jar with -glm BOTH -T -stand_call_conf 50.0 -dcov 1000 -A Coverage -A AlleleBalance called the variants of SNPs and small indels (1–50 bp). The SNPs of population filter using the hard filter parameters: a MQ0 ≥ 4 && ((MQ0 / (1.0 * DP)) > 0.1); b DP < 5; c QUAL < 30.0; d QUAL > 30.0 && QUAL < 50.0; e QD < 1.5; according to the official software instructions of GATK (https://gatk.broadinstitute.org/hc/en-us/articles/360035890471-Hard-filtering-germline-short-variants). Since there were no SNPs of *L. corniculatus* available before our sequencing, we chose Hard Filtering after best practice tutorial.

### Measurement of cyanogenic glycosides content

The top leaves in full-bloom stage were harvested, frozen, and lyophilized. Samples were smashed by Tissue Lyser and kept at − 20 °C. Samples (10 mg) were extracted with 10 ml 85% v/v methanol and boiled in 65 °C water bath for 5 min, followed by cooling on ice. The solution was then filtered through the 0.22-μm organic microporous filter. Analytical LC–MS was performed using Agilent G6500 Series HPLC-QQQ and A Zorbax SB-C18 column (Agilent; 1.8 mM, 2.1 × 75 mm). The flow rate used as previous study; MS/MS analysis was performed as described elsewhere [51, 60]. The mass spectrometer was run in positive electrospray mode.

### Phylogenetic and population structure analyses

All the filtered SNPs were used for phylogenetic and population structure analyses. Phylogenetic tree was constructed by Phylip v3.696 based on NJ (neighbor-joining) method and the result was displayed by iTOL (https://itol.embl.de/). EIGENSOFT v6.0.1 [61] and Admixture v1.3.0 [60] were used for principal components analysis (PCA) and population structure analysis, respectively. The linkage disequilibriums (LD) analysis was performed by PopLDdecay v1.29 (https://github.com/BGI-shenzhen/PopLDdecay).

### Selective sweep and nucleotide diversity Analyses

We used Vcftools v0.1.14 [62] to calculate *Fst* and PI, the selected sweep analysis was conducted with 10 Kb windows sliding at 5 Kb steps. Regions with top 5% values of *Fst* values were recognized as candidate regions. The figure results were produced by RectChr-1.31 (https://github.com/BGI-shenzhen/RectChr).

### GWAS and identification of the candidate genes

The SNPs with MAF ≥ 0.05 and missing rate ≤ 0.1in 241 *L. corniculatus* accessions (Group II and Group III) were used for GWAS of 3 traits. The multi-locus mixed model (MLMM) [63], fixed and random model circulating probability unification (FarmCPU) [64], and Bayesian-information and linkage-disequilibrium iteratively nested keyway (BLINK) [65] program were used for GWAS analyses by GAPIT R package. According to the LD decay of Group II and Group III (Fig. S1c), upstream and downstream 30 kb region of associated loci was determined. The *p* value (*p* < 2.0 × 10^–5^) was defined as the genome-wide significance threshold.

### RNA extraction, cDNA synthesis, and quantitative PCR

For RNA extraction, the *L. japonicus* seeding and leaves were collected 1.5 month after planting, the top leaves of *L. corniculatus* accessions were collected during full-bloom stage from the field in Beijing and leaves of *Arabidopsis thaliana* were collected 4 weeks after planting. RNApre Pure Plant Plus Kit (DP441, Tiangen, Beijing, China) were used for total RNA extraction. For cDNA synthesis, HiScript III RT SuperMix for qPCR (R323-01, Vazyme, Nanjing, China) was used according to the manufacturer’s protocol. The qRT-PCR was performed as the protocol of ChamQ Universal SYBR qPCR Master Mix (Q711, Vazyme, Nanjing, China). The primers used for qPCR are listed in Table S15.

### Site-directed mutagenesis

The 230 base G of *LjMTR* CDS was mutated into C (*LjMTR*-D230), and 87 base G upstream of *LjZCA* was mutated into T (Pro-ZCA-D87) using Fast Site-Directed Mutagenesis Kit (KM101, TIANGEN BIOTECH, Beijing, China) according to the manufacturer’s protocol.

### Dual luciferase transactivation assay

The promoters of *CYP79D3*, *CYP736A2*, *UGT85k3*, and Pro-ZCA, Pro-ZCA-D87 were cloned into pGreenII 0800-LUC reporter vector. Full length of *LjZCD*, *LjMTR,* and *LjMTR*-D230 were cloned into pRT101 acting as the effector [66]. The pRT101 empty vector was used as control. All primer sequences are listed in Table S1.

For protoplasts transactivation, leaves of 4-week *Arabidopsis thaliana* were harvested for protoplasts isolation. The methods of protoplasts isolation and co-transformation were according to the previous study [67]. The 5 µg reporter vector and 5 µg effector plasmids were co-transformed into *Arabidopsis* protoplasts. Protoplasts were harvested and snap frozen in liquid nitrogen 16 h after transformation. The Dual-Luciferase® Reporter Assay kit was used for luciferase activity (E1910, Promega) according to manufacturer’s protocol. Values represent the mean ± SE of 4 repeated experiments.

### Subcellular location

The *LjMTR*, *LjZCD,* and *LjZCB* were fused with GFP into pAN580-GFP for subcellular localization. The pBSK-35S-H2B-Mcherry vector used as the nuclear localization maker (jxb). All primer sequences are displayed in Supplementary Table S1. The *Arabidopsis* protoplasts transactivation performed the same as mentioned above. All the photos were taken by Zeiss LSM980 confocal microscope. The GFP signal was detected by an argon laser line of 488 nm (excitation) and a band pass emission filter of 505–530 nm. To visualize the Mcherry signal, a 543-nm laser and a 560/615-nm band pass filter were used.

### Establishment of overexpressed materials

The CDS of *LjZCD*, *LjZCB*, and *LjZCA* were inserted into pCambia1307 fused with 5 × MYC label. *Agrobacterium* strain EHA105 with pCambia1307-35S-5xMYC-*LjZCD* was used for *L. japonicus* transformation. The *Agrobacterium tumefaciens* strain GV3101 with pCambia1307-35S-5xMYC-*LjZCB* and pCambia1307-35S-5xMYC-*LjZCA* were used for *Arabidopsis* transformation.

For *L. japonicus* transformation, *L. japonicus* accession Miyakojima MG-20 was used for hypocotyl transformation. The medium and operation of hypocotyl transformation and regeneration were according to a previous report [31]. For *Arabidopsis* transformation, MS + 5% sucrose + 0.05% Silwet L-77 solution was used for *A. tumefaciens* GV3101 resuspension. The agrobacterial solution dipped *Arabidopsis* flowers for 5 min, plants were kept in the dark approximately 18 h after dipping and grown until seeds were harvested. T1 seeds were sown on MS media containing 20 mg/L Hygromycin B for selecting transgenic plants. The grown condition of all plants was 24 °C and the light period was a 16-h-light/8-h-dark regime.

All the overexpressed materials were identified by PCR and qPCR using DNA and cDNA respectively. All primer sequences are displayed in Table S16.

### Statistical analysis

Two-tailed Student’s *t*-test and one-way ANOVA were used for the data analysis. Significant differences between values are indicated with different letters (*, *P* < 0.05; **, *P* < 0.005; ***, *P* < 0.0001).

### Supplementary Information

**Additional file 1: Table S1.** Basic information of 272 *L. corniculatus* accessions. **Table S2.** The Cross Validation error of values for the K values. **Table S3.** Selective sweeps between Group I and Group II. **Table S4.** Selective sweeps between Group I and Group III. **Table S5.** Selective sweeps between Group II and Group III. **Table S6.** Genes located in selective sweeps between Group I and Group II. **Table S7.** Genes located in selective sweeps between Group I and Group III. **Table S8.** Genes located in selective sweeps between Group II and Group III. **Table S9.** The CNglcs content and growth traits of 272 *L. corniculatus* accessions. **Table S10.** Total CNglcs content associated genes identified by GWAS on chromosome 6: 22.28—22.34 Mb. **Table S11.** Stem length associated genes identified by GWAS on chromosome 5: 19.86–19.92 Mb in May 2020 BeiJing. **Table S12.** Steam length associated genes identified by GWAS on chromosome 2: 34.67–34.73 Mb in May 2021 LiangShan. **Table S13.** Plant height associated genes identified by GWAS on chromosome 3: 6.76–7.36 Mb in September 2019 BeiJing. **Table S14.** Plant height associated genes identified by GWAS on chr:3 11.20–11.26 Mb in September LiangShan. **Table S15.** Primers for q-PCR. **Table S16.** Primers used for vector constructs.

**Additional file 2: Fig. S1. a** Neighbor-joining tree of 273 germplasms, including 272 *L. corniculatus* accessions and 1 *L. frondosus*. **(b-c)** Linkage disequilibrium (LD) decay distance of *L. corniculatus* groups. **d** Neighbor-joining tree of 274 germplasms, including 272 *L. corniculatus* accessions, *L. frondosus* and *L. japonicus*. **Fig. S2. a**, **b**, **c** Box plot of plant height, stem length and CNglcs content in *L. corniculatus* groups. Significant differences between values are indicated with different letters (*, *P* < 0.05; **, *P* < 0.005; ***, *P* < 0.0001). **d** Bar plots of Go enrichment of selective-sweep signals identified genes through comparisons between Group I and Group II (upper panel), Group I and Group III (middle panel), and spring and winter ecotypes (lower panel). **Fig. S3-5.** Manhattan plots for total CNglcs content, lotaustraline and linamarin in 241 accessions using MLMM, Blink and FarmCPU. The black dashed lines indicate the significance threshold (*p* value = 2.0 × 10^–5^) and black arrow indicates the significant GWAS peak. **Fig. S6. a** Relative expression of CNglcs synthetic genes *CYP79D3*, *CYP736A2* and *UGT85K3* in different accessions carrying Hap.G and Hap.S, respectively. **b** Expression profile of CNglcs related genes in Hap.G and Hap.S. **Fig. S7.** Identification of overexpressed materials in *Arabidopsis* and *L. corniculatus*. **a** PCR identification of positive transformed plants of 35S::*ZCD* in *L. corniculatus*. **b** Relative expression of *LjZCD* in *WT* and overexpressed *LjZCD* plants of *L. corniculatus*. **c** PCR identification of positive transformed plants of 35S::*ZCB* in *Arabidopsis*. **d** Relative expression of *LjZCB* in *WT* and overexpressed *LjZCB* plants of *Arabidopsis*. **Fig. S8-12.** Manhattan plots for stem length in 241 accessions using MLMM, Blink and FarmCPU. The black dashed lines indicate the significance threshold (*p* value = 2.0 × 10^–5^) and black arrow indicates the significant GWAS peak. **Fig. S13.** Expression profile of different haplotype. **a** stem length related genes in Hap.G and Hap.K. **b** Expression profile of plant height related genes in Hap.C and Hap.Y. **Fig. S14-18.** Manhattan plots for plant height in 241 accessions using MLMM, Blink and FarmCPU. The black dashed lines indicate the significance threshold (*p* value = 2.0 × 10^–5^) and black arrow indicates the significant GWAS peak.

## Data Availability

All genomic sequence raw data sets for genetic diversity analysis and GWAS are available from the National Center for Biotechnology Information (https://www.ncbi.nlm.nih.gov/) under BioProject accession no. PRJNA996875 [68].

## Abbreviations

SNP: Single nucleotide polymorphism
CNglcs: Cyanogenic glycosides
PCA: Principal component analysis
LD: Linkage disequilibrium
*Fst*: Population fixation statistics
GWAS: Genome-wide associated study
MLMM: Multi-locus mixed model
FarmCPU: Fixed and random model circulating probability unification
BLINK: Bayesian-information and Linkage-disequilibrium iteratively nested keyway
PH: Plant height
SL: Stem length
*L. corniculatus*: *Lotus corniculatus*
*T. pratense*: *Trifolium pratense*
*M. sativa*: *Medicago sativa*
*L. japonicus*: *Louts japonicus*
*L. frondosus*: *Lotus frondosus*
*A. tumefaciens*: *Agrobacterium tumefaciens*
