# Supplementary material for: Resequencing of global Lotus corniculatus accessions reveals population distribution and genetic loci, associated with cyanogenic glycosides accumulation and growth traits
Source: BMC Biol. 2023 Aug 17;21:176. doi: 10.1186/s12915-023-01670-7 (PMC10433565; doi:10.1186/s12915-023-01670-7)
Supplement: Supplementary file 2 — Additional file 2: Fig. S1. a Neighbor-joining tree of 273 germplasms, including 272 L. corniculatus accessions and 1 L. frondosus. (b-c) Linkage disequilibrium (LD) decay distance of L. corniculatus groups. d Neighbor-joining tree of 274 germplasms, including 272 L. corniculatus accessions, L. frondosus and L. japonicus. Fig. S2. a, b, c Box plot of plant height, stem length and CNglcs content in L. corniculatus groups. Significant differences between values are indicated with different letters (*, P < 0.05; **, P < 0.005; ***, P < 0.0001). d Bar plots of Go enrichment of selective-sweep signals identified genes through comparisons between Group I and Group II (upper panel), Group I and Group III (middle panel), and spring and winter ecotypes (lower panel). Fig. S3-5. Manhattan plots for total CNglcs content, lotaustraline and linamarin in 241 accessions using MLMM, Blink and FarmCPU. The black dashed lines indicate the significance threshold (p value = 2.0 × 10^–5^) and black arrow indicates the significant GWAS peak. Fig. S6. a Relative expression of CNglcs synthetic genes CYP79D3, CYP736A2 and UGT85K3 in different accessions carrying Hap.G and Hap.S, respectively. b Expression profile of CNglcs related genes in Hap.G and Hap.S. Fig. S7. Identification of overexpressed materials in Arabidopsis and L. corniculatus. a PCR identification of positive transformed plants of 35S::ZCD in L. corniculatus. b Relative expression of LjZCD in WT and overexpressed LjZCD plants of L. corniculatus. c PCR identification of positive transformed plants of 35S::ZCB in Arabidopsis. d Relative expression of LjZCB in WT and overexpressed LjZCB plants of Arabidopsis. Fig. S8-12. Manhattan plots for stem length in 241 accessions using MLMM, Blink and FarmCPU. The black dashed lines indicate the significance threshold (p value = 2.0 × 10^–5^) and black arrow indicates the significant GWAS peak. Fig. S13. Expression profile of different haplotype. a stem length related genes in Hap.G and Hap.K [file 12915_2023_1670_MOESM2_ESM.pdf]

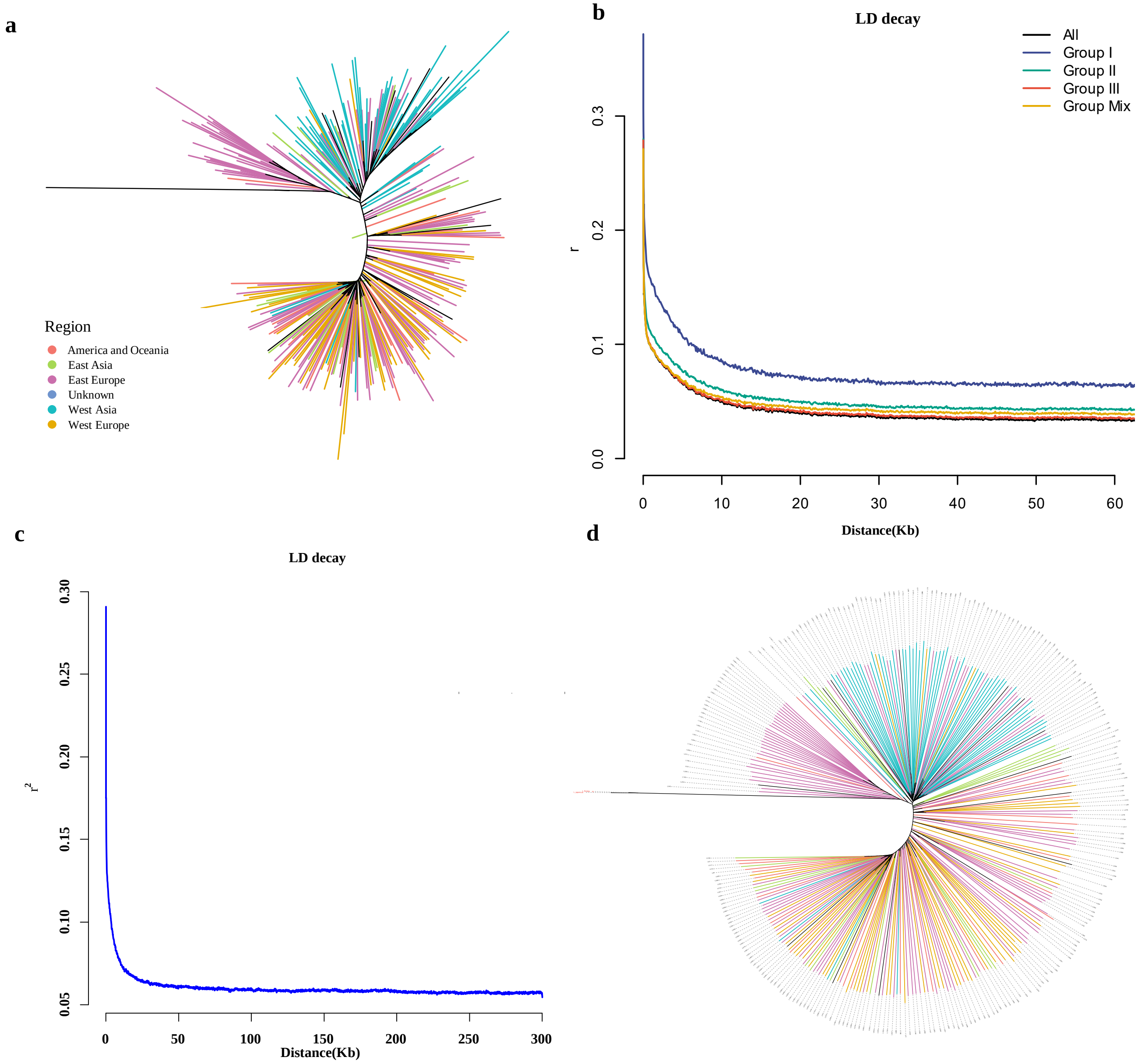

Fig. S1 **a** Neighbor-joining tree of 273 germplasms, including 272 *L. corniculatus* accessions and 1 *L. frondosus*. **b-c** Linkage disequilibrium (LD) decay distance of *L. corniculatus* groups. **d** Neighbor-joining tree of 274 germplasms, including 272 *L. corniculatus* accessions, *L. frondosus* and *L. japonicus*.

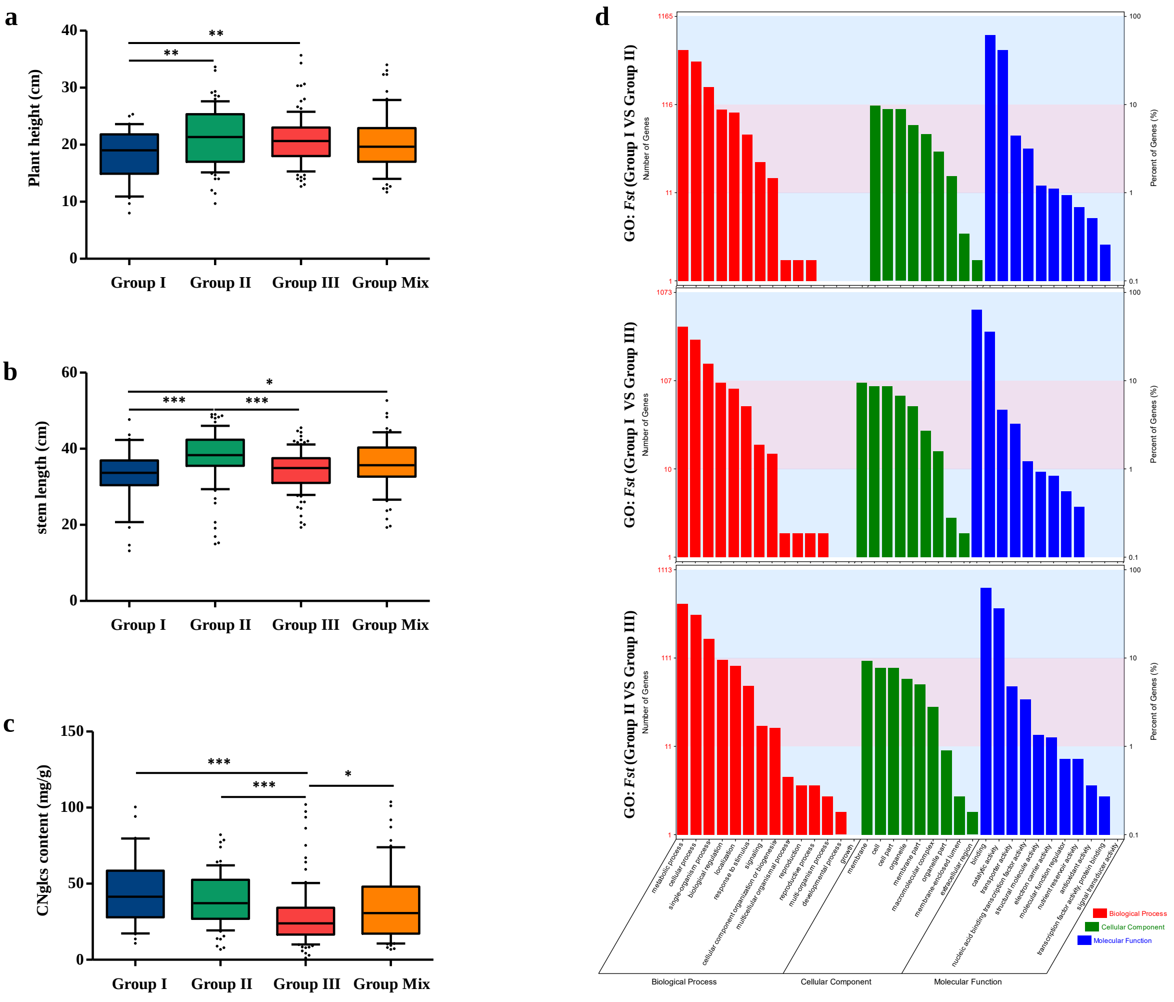

Fig. S2 a, **b**, **c** Box plot of plant height, stem length and CNglcs content in *L. corniculatus* groups. Significant differences between values are indicated with different letters (*, P<0.05; **, P<0.005; ***, P<0.0001). **d** Bar plots of Go enrichment of selective-sweep signals identified genes through comparisons between Group I and Group II (upper panel), Group I and Group III (middle panel), and spring and winter ecotypes (lower panel).

MLMM-Total CNglcs

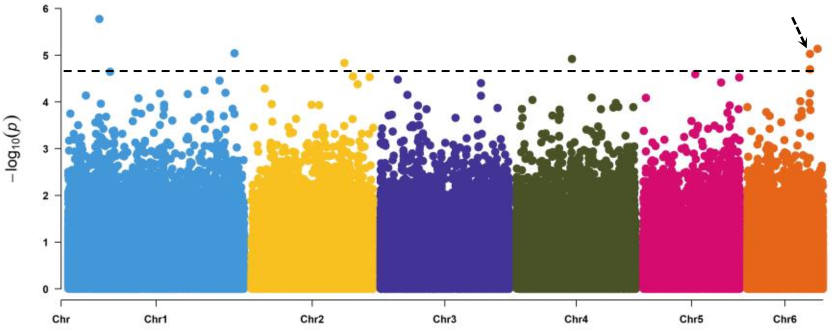

Blink-Total CNglcs

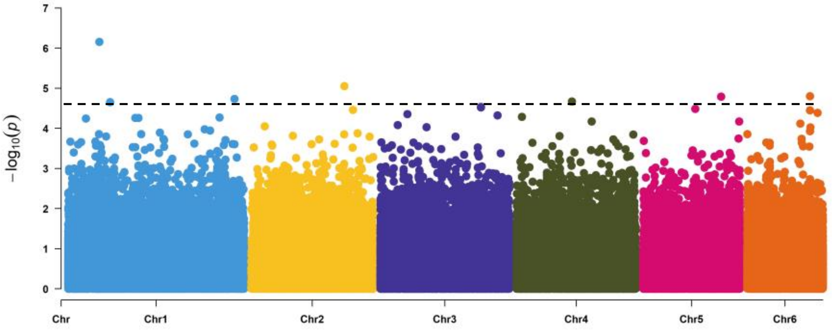

FarmCPU-Total CNglcs

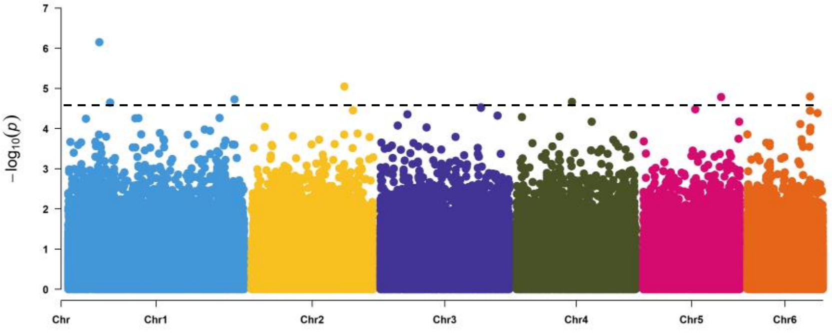

Fig. S3 Manhattan plots for total CNglcs content of 241 accessions using MLMM, Blink and FarmCPU. The black dashed lines indicate the significance threshold (p value = 2.0 x 10^{-5}) and black arrow indicates the significant GWAS peak.

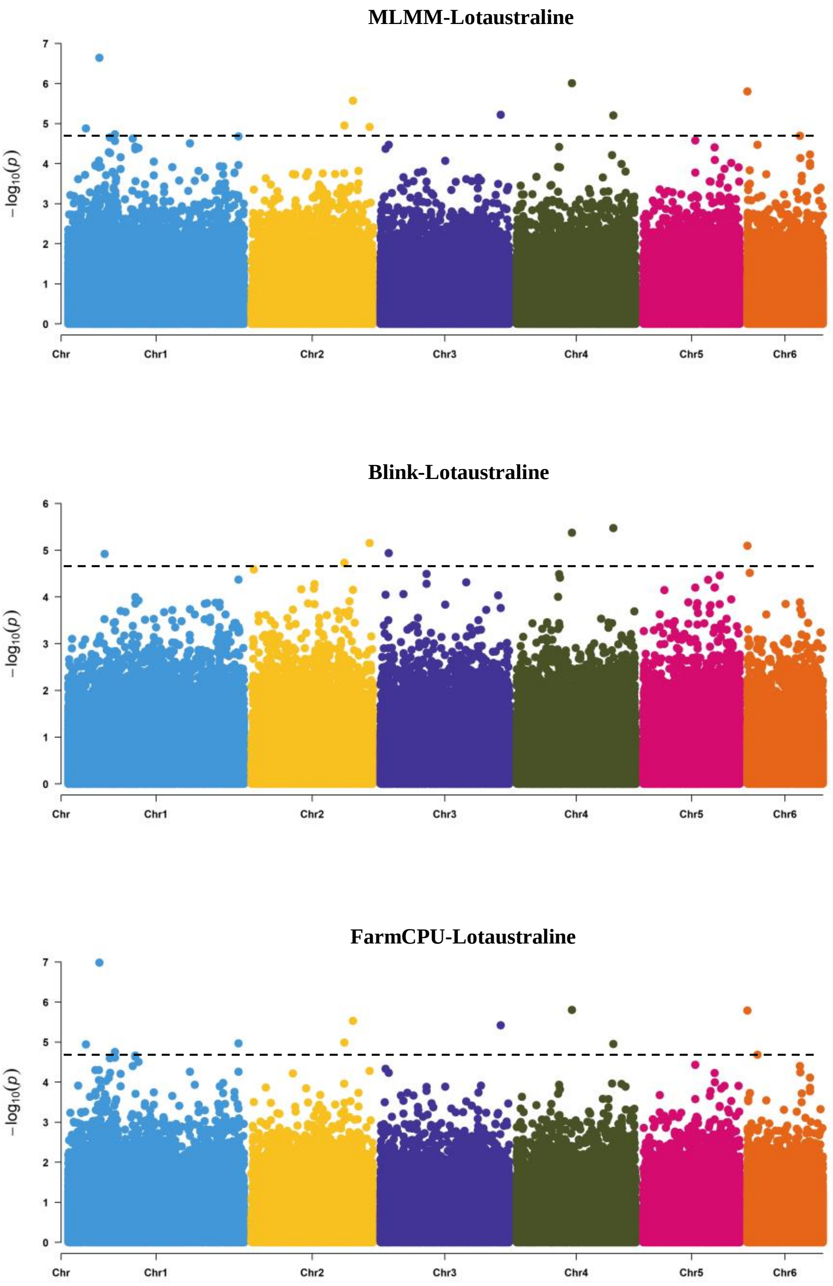

Fig. S4 Manhattan plots for lotaustraline of 241 accessions using MLMM, Blink and FarmCPU. The black dashed lines indicate the significance threshold (p value = 2.0×10^{-5}).

MLMM-Linamarin

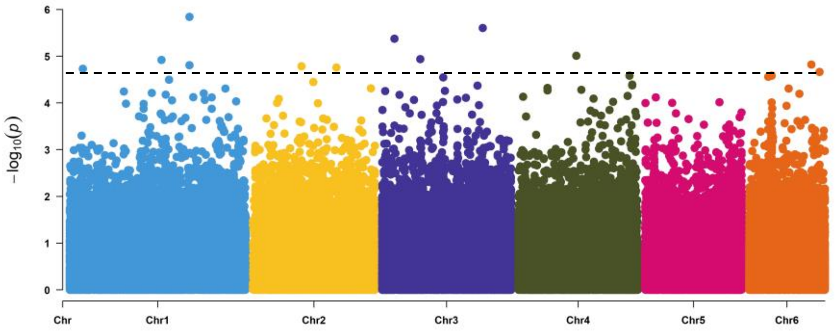

Blink-Linamarin

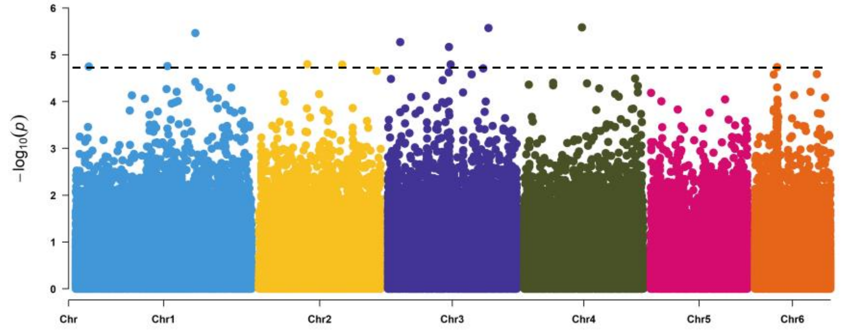

FarmCPU-Linamarin

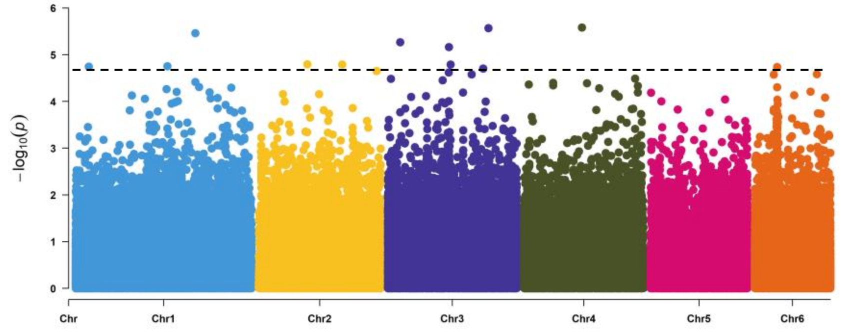

Fig. S5 Manhattan plots for linamarin of 241 accessions using MLMM, Blink and FarmCPU. The black dashed lines indicate the significance threshold (p value = 2.0×10^{-5}).

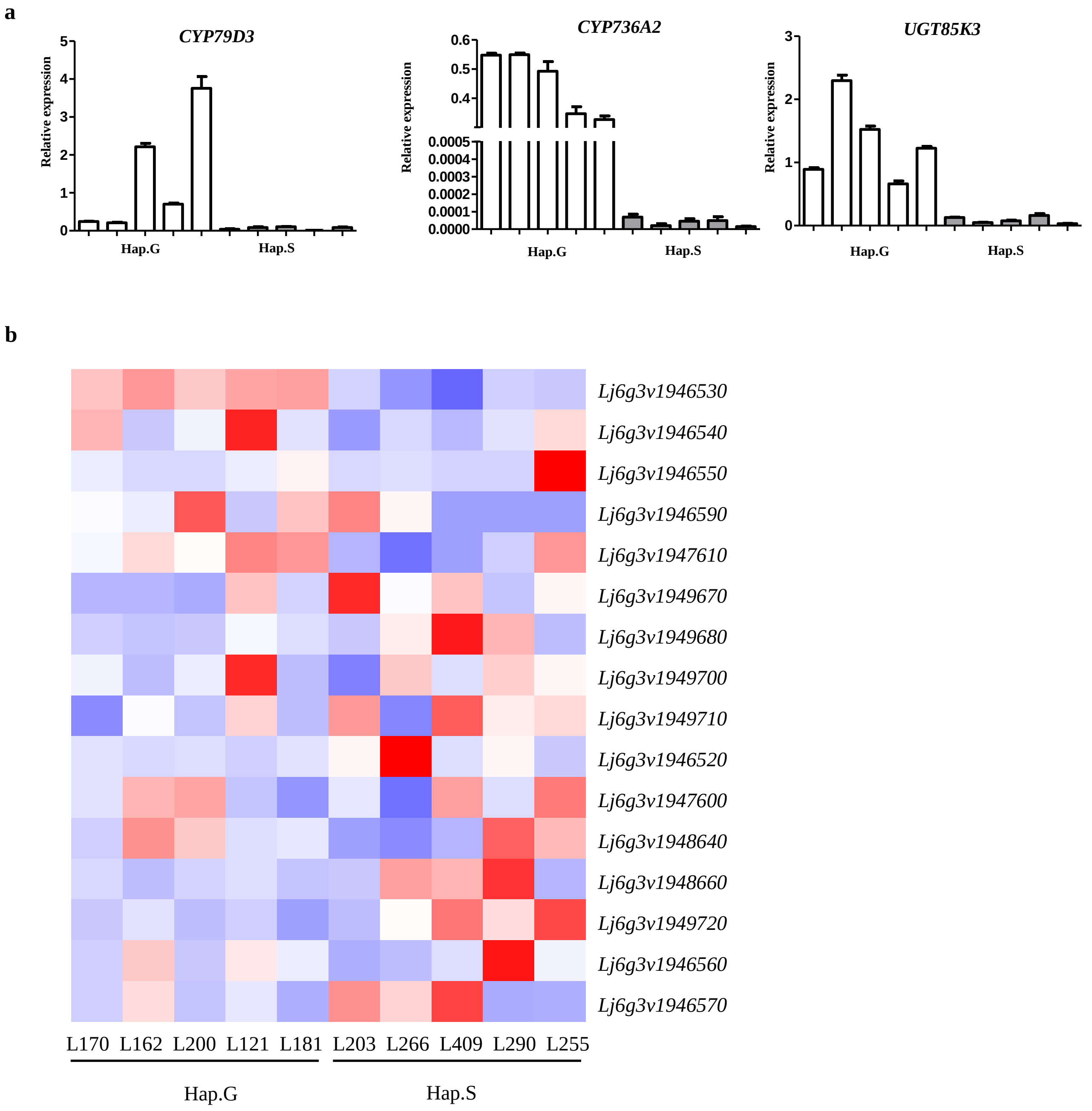

Fig. S6 a Relative expression of CNglcs synthetic genes *CYP79D3*, *CYP736A2* and *UGT85K3* in different accessions carrying Hap.G and Hap.S, respectively. **b** Expression profile of CNglcs related genes in Hap.G and Hap.S.

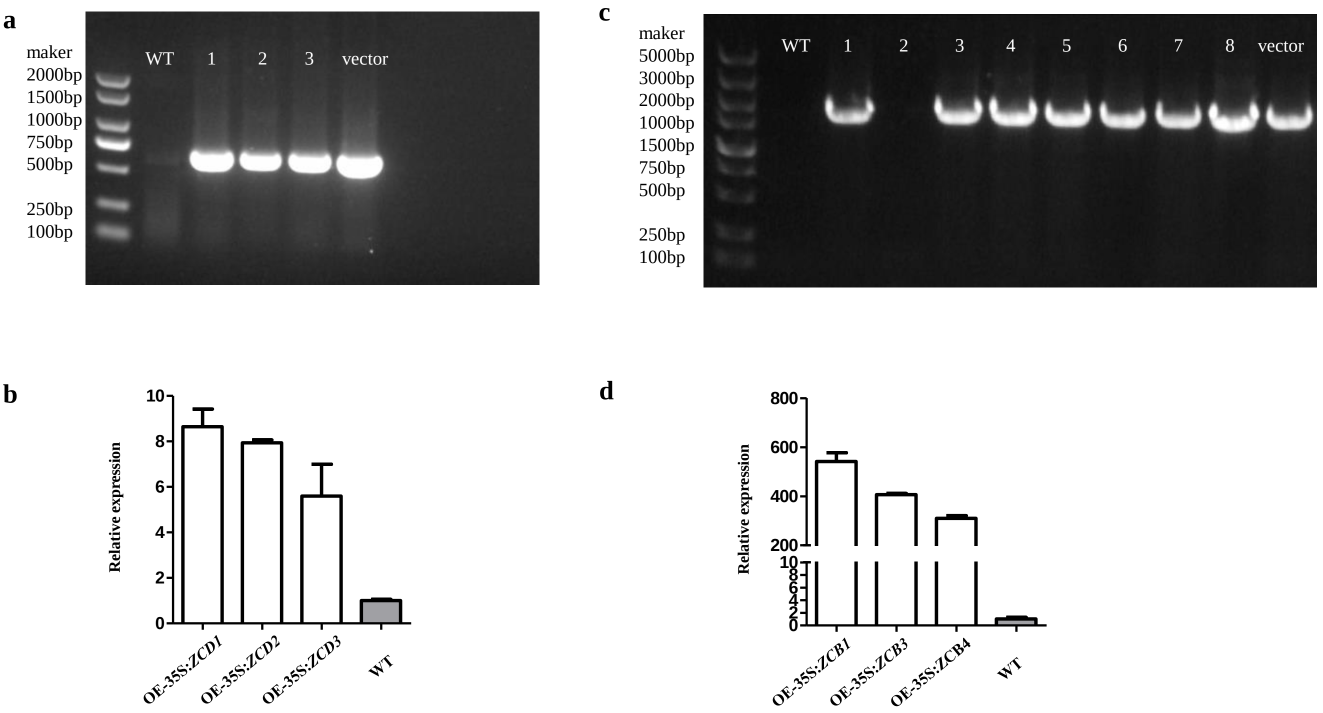

Fig. S7 Identification of overexpressed materials in *Arabidopsis* and *L. corniculatus.* **a** PCR identification of positive transformed plants of OE-35S:*ZCD* in *L. corniculatus*. **b** Relative expression of *LjZCD* in wild type (WT) and overexpressed *LjZCD* plants of *L. corniculatus*. **c** PCR identification of positive transformed plants of OE-35S:*ZCB* in *Arabidopsis.* **d** Relative expression of *LjZCB* in wild type (WT) and overexpressed *LjZCD* plants of *Arabidopsis*.

MLMM-SL_Jul.19B

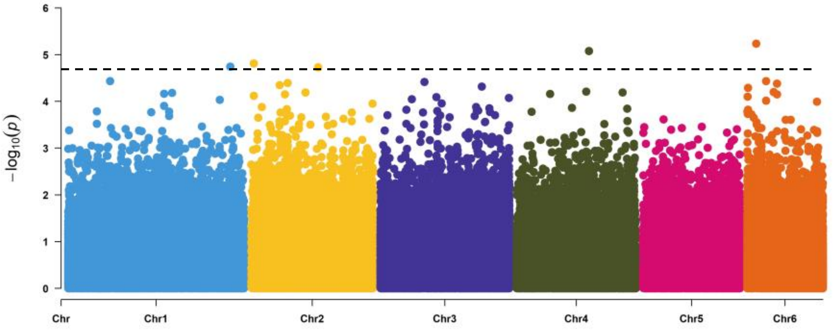

Blink-SL_Jul.19B

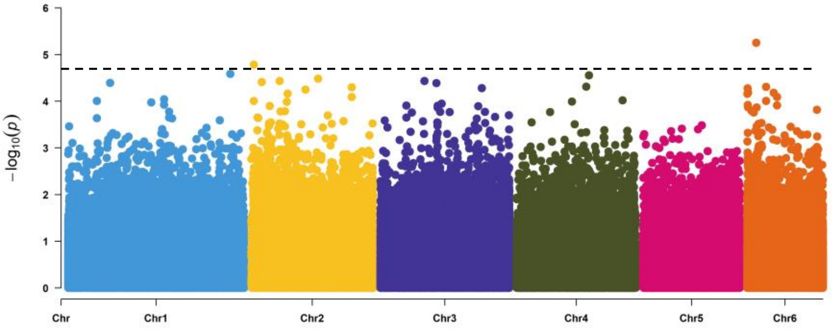

FarmCPU-SL_Jul.19B

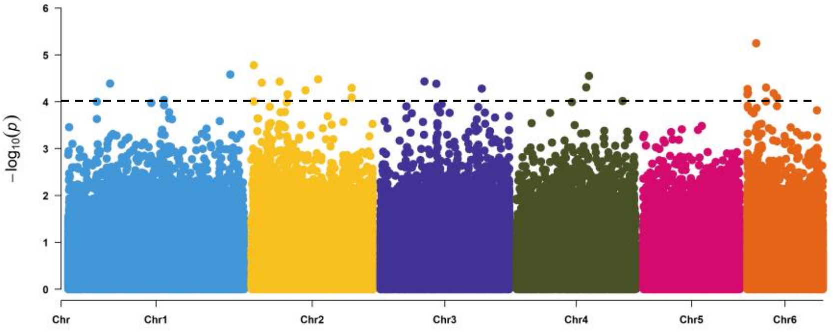

Fig. S8 Manhattan plots for stem length of 241 accessions in July 2019 BeiJing using MLMM, Blink and FarmCPU. The black dashed lines indicate the significance threshold (p value = 2.0 x 10^{-5}).

MLMM-SL_Sep.19B

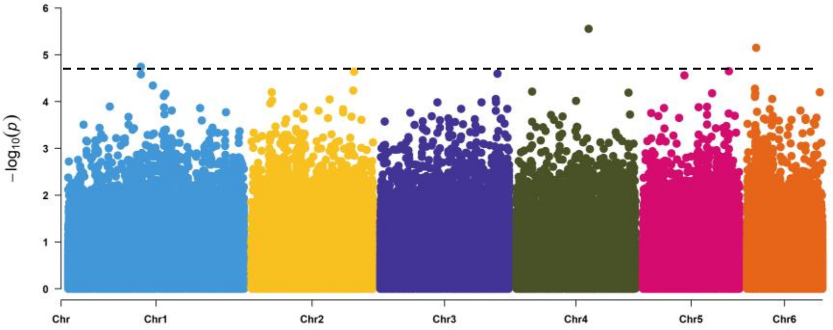

Blink-SL_Sep.19B

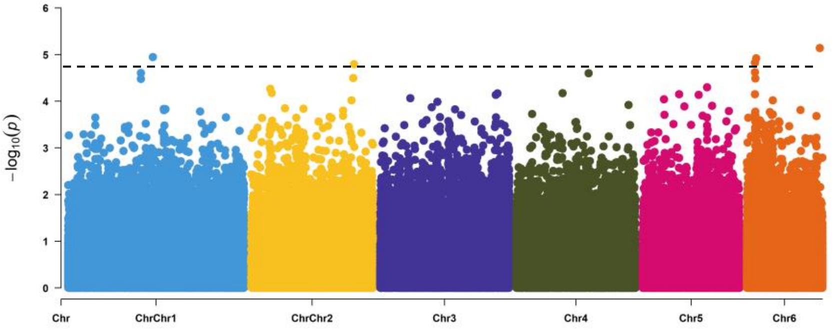

FarmCPU-SL_Sep.19B

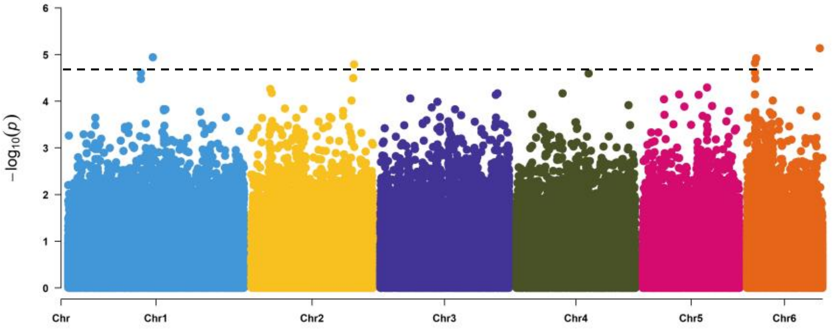

Fig. S9 Manhattan plots for stem length of 241 accessions in September 2019 BeiJing using MLMM, Blink and FarmCPU. The black dashed lines indicate the significance threshold (p value = 2.0 x 10^{-5}).

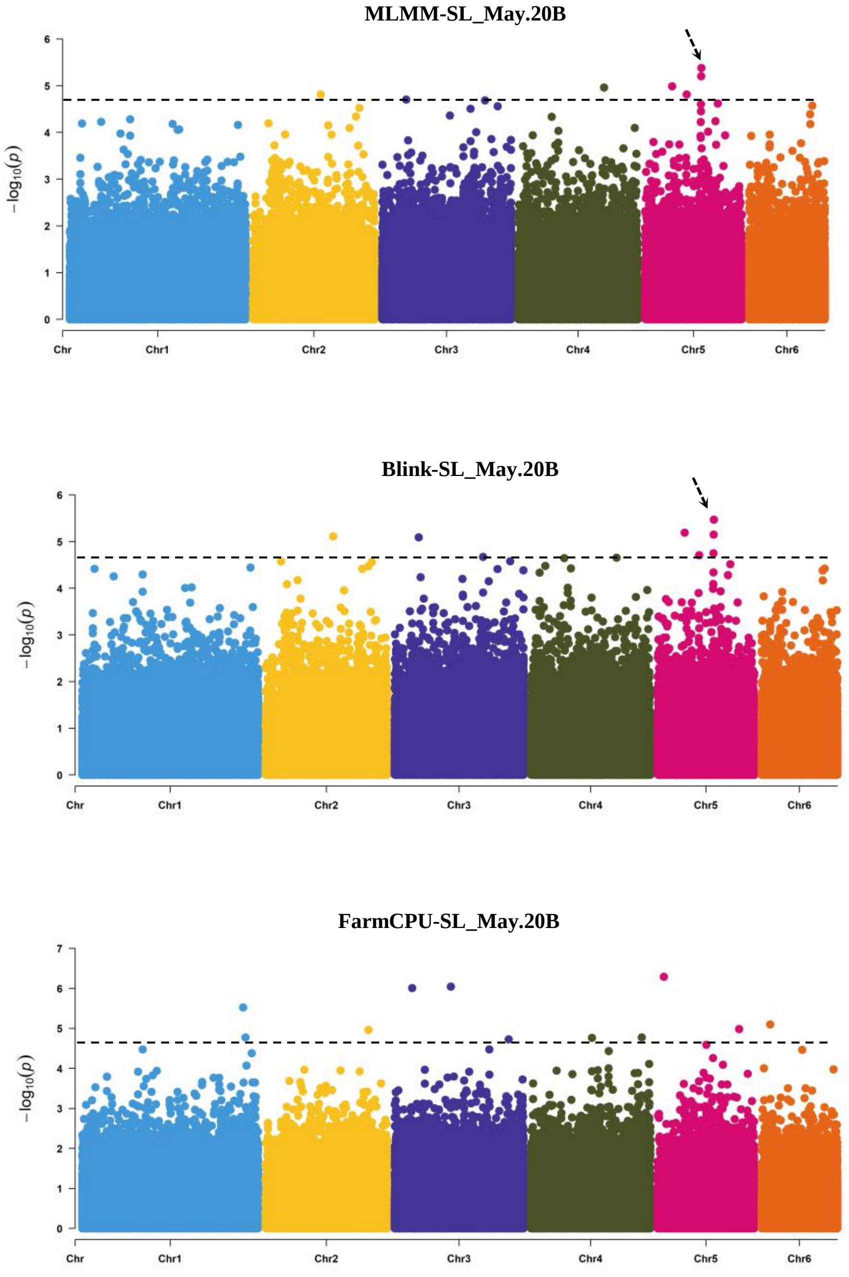

Fig. S10 Manhattan plots for stem length of 241 accessions in May 2020 BeiJing using MLMM, Blink and FarmCPU. The black dashed lines indicate the significance threshold (p value = 2.0×10^{-5}) and black arrow indicates the significant GWAS peak.

MLMM-SL_Sep.20L

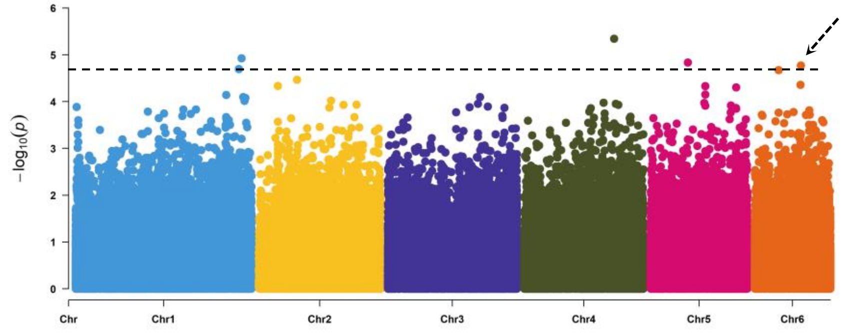

Blink-SL_Sep.20L

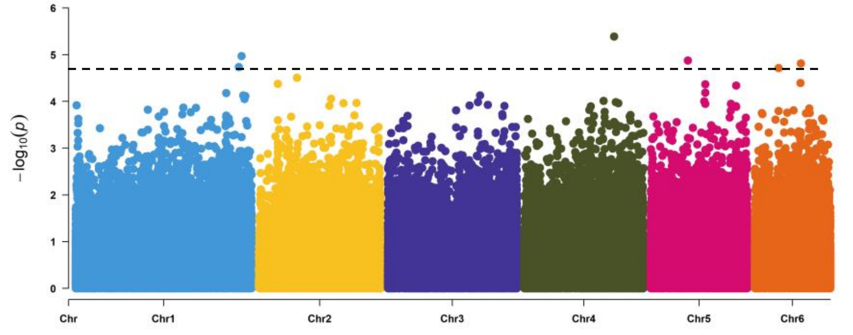

FarmCPU-SL_Sep.20L

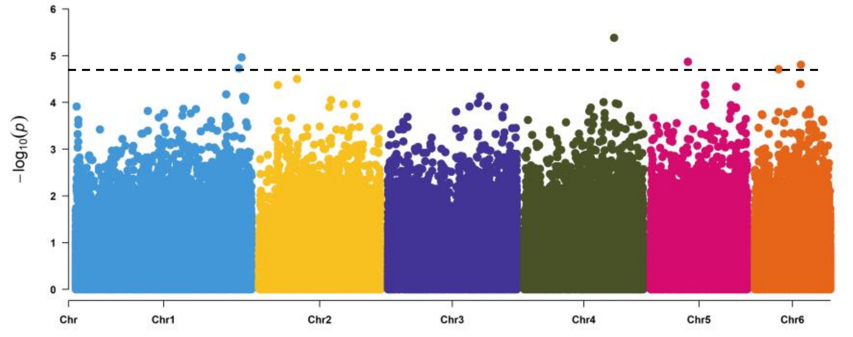

Fig. S11 Manhattan plots for stem length of 241 accessions in September 2020 LiangShan using MLMM, Blink and FarmCPU. The black dashed lines indicate the significance threshold (p value = 2.0 x 10^{-5}) and black arrow indicates the significant GWAS peak.

MLMM-SL_May.21L

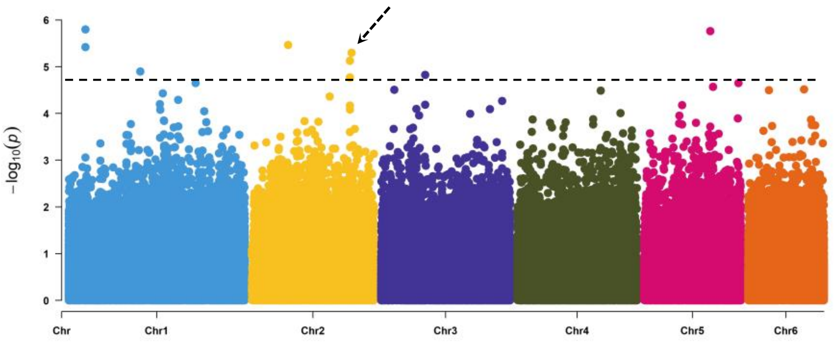

Blink-SL_May.21L

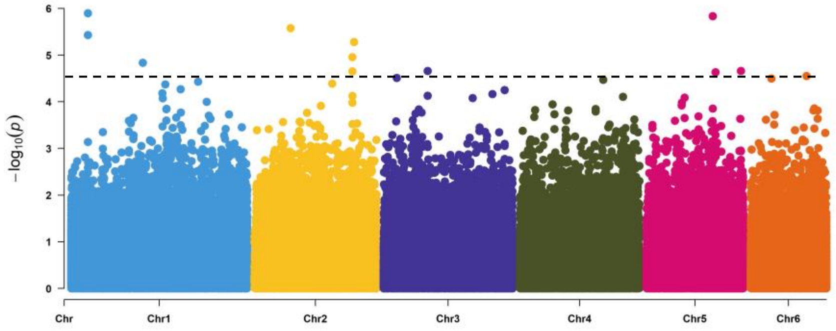

FarmCPU-SL_May.21L

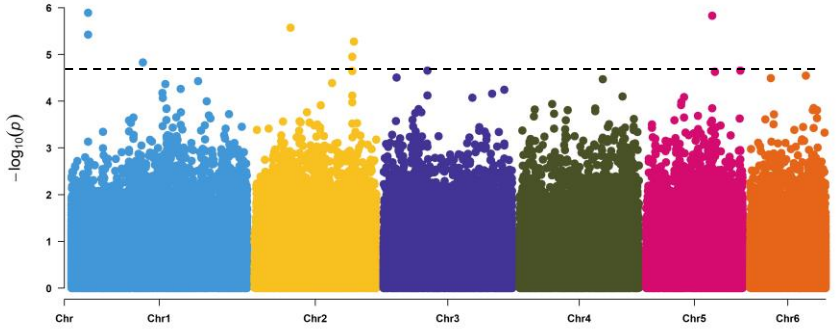

Fig. S12 Manhattan plots for stem length of 241 accessions in May 2021 LiangShan using MLMM, Blink and FarmCPU. The black dashed lines indicate the significance threshold (p value = 2.0 x 10^{-5}) and black arrow indicates the significant GWAS peak.

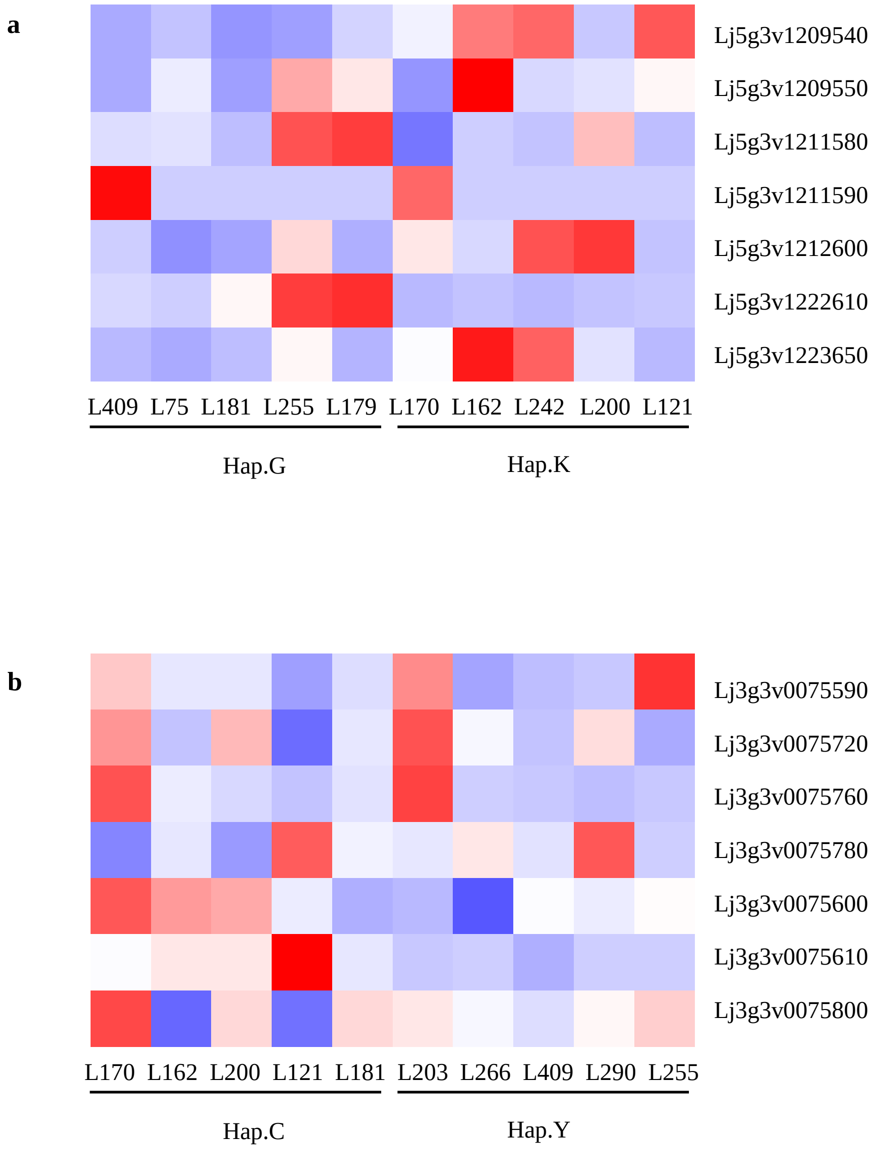

Fig. S13 a Expression profile of stem length related genes in Hap.G and Hap.K. **b** Expression profile of plant height related genes in Hap.C and Hap.Y.

MLMM-PH_Jul.19B

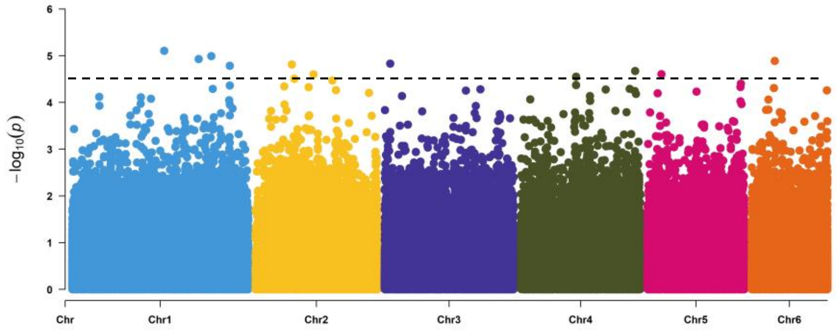

Blink-PH_Jul.19B

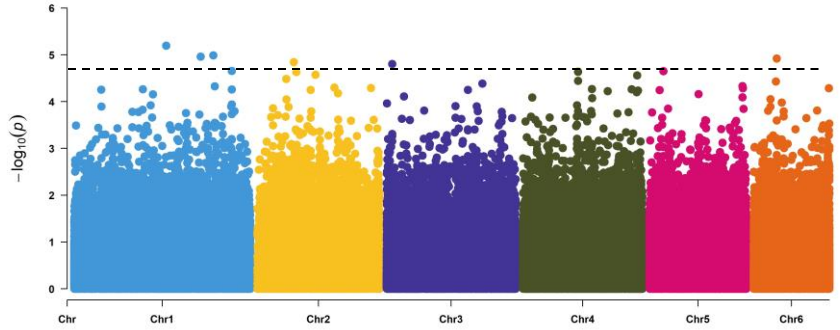

FarmCPU-PH_Jul.19B

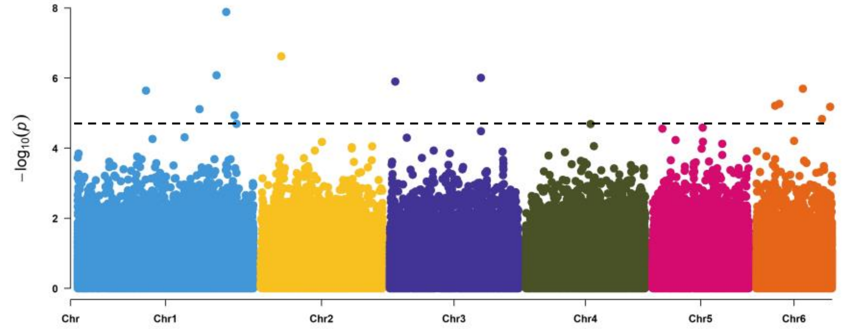

Fig. S14 Manhattan plots for plant height of 241 accessions in July 2019 BeiJing using MLMM, Blink and FarmCPU. The black dashed lines indicate the significance threshold (p value = 2.0×10^{-5}).

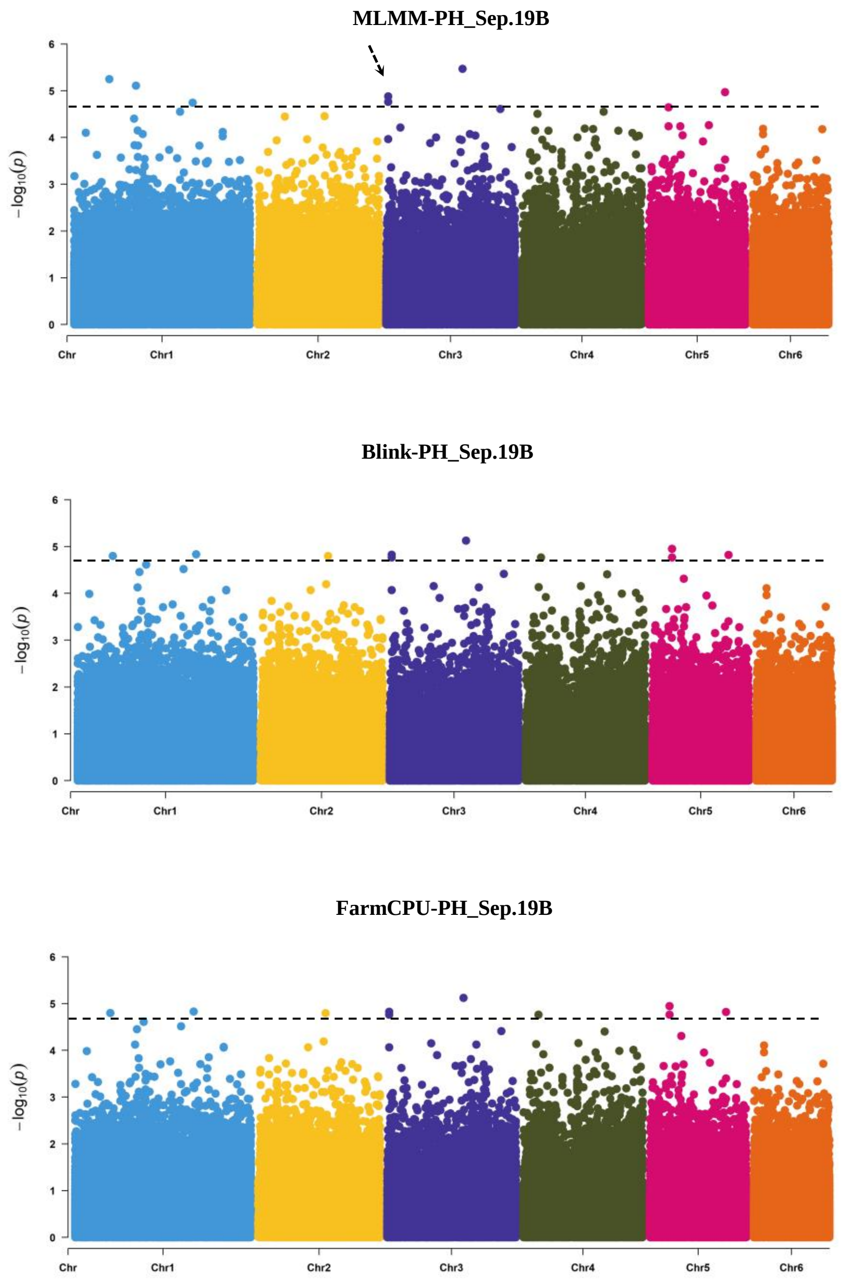

Fig. S15 Manhattan plots for plant height of 241 accessions in September 2019 BeiJing using MLMM, Blink and FarmCPU. The black dashed lines indicate the significance threshold (p value = 2.0 x 10^{-5}) and black arrow indicates the significant GWAS peak.

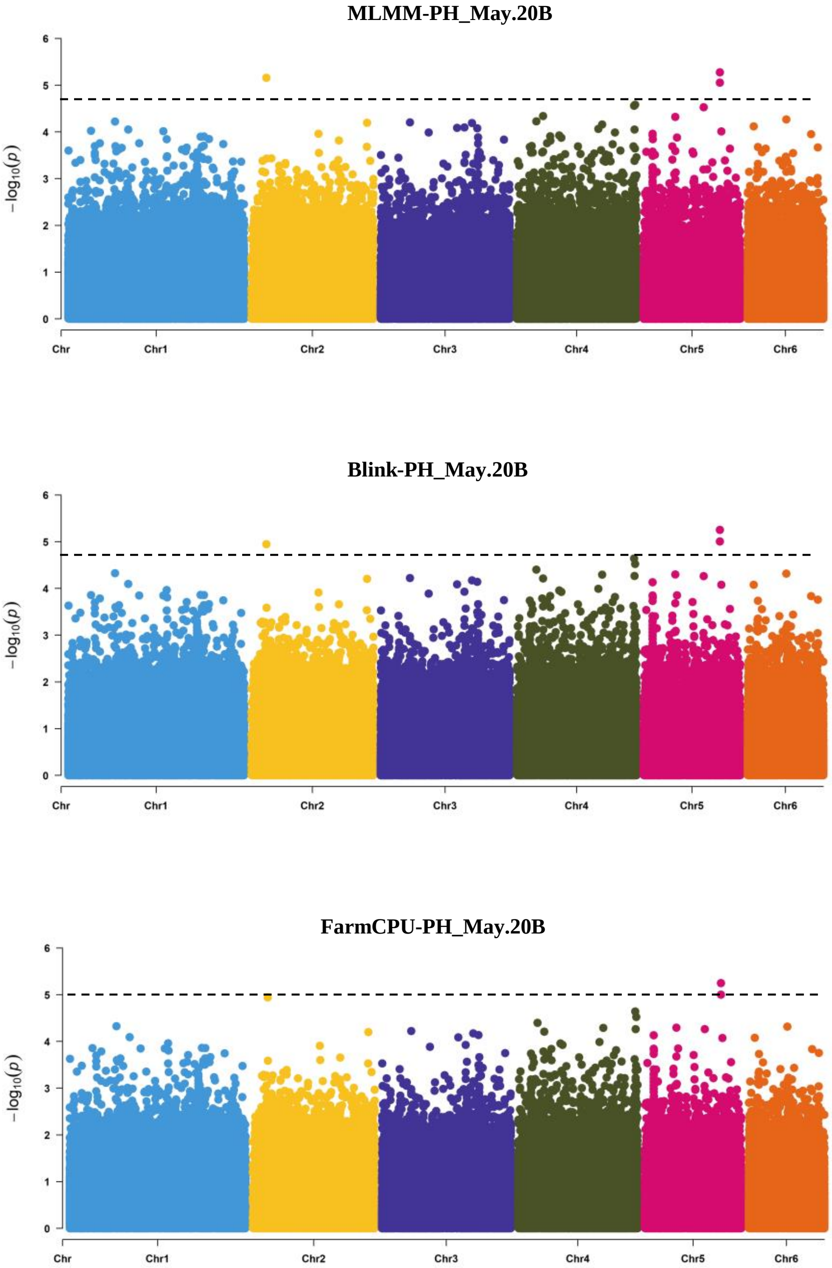

Fig. S16 Manhattan plots for plant height of 241 accessions in May 2020 BeiJing using MLMM, Blink and FarmCPU. The black dashed lines indicate the significance threshold (p value = 2.0×10^{-5}).

MLMM-PH_Sep.20L

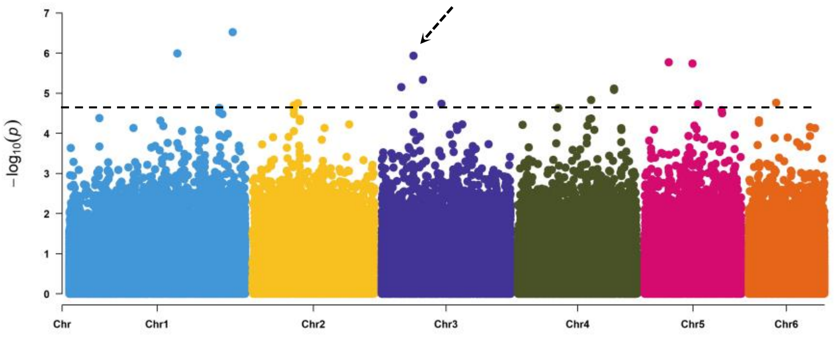

Blink-PH_Sep.20L

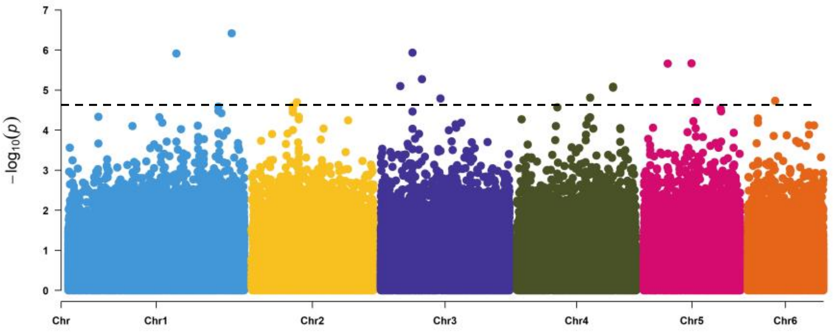

FarmCPU-PH_Sep.20L

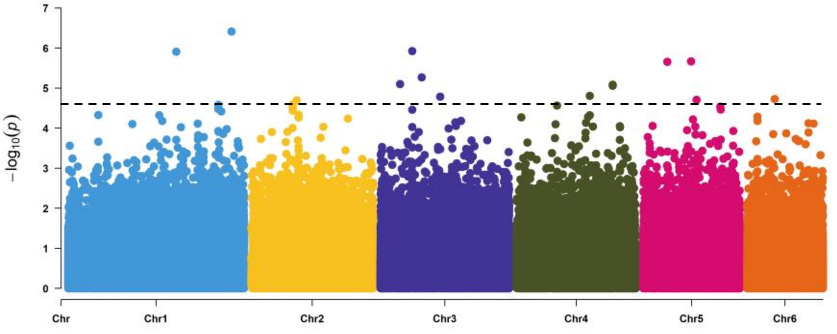

Fig. S17 Manhattan plots for plant height of 241 accessions in September 2020 LiangShan using MLMM, Blink and FarmCPU. The black dashed lines indicate the significance threshold (p value = 2.0 x 10^{-5}) and black arrow indicates the significant GWAS peak.

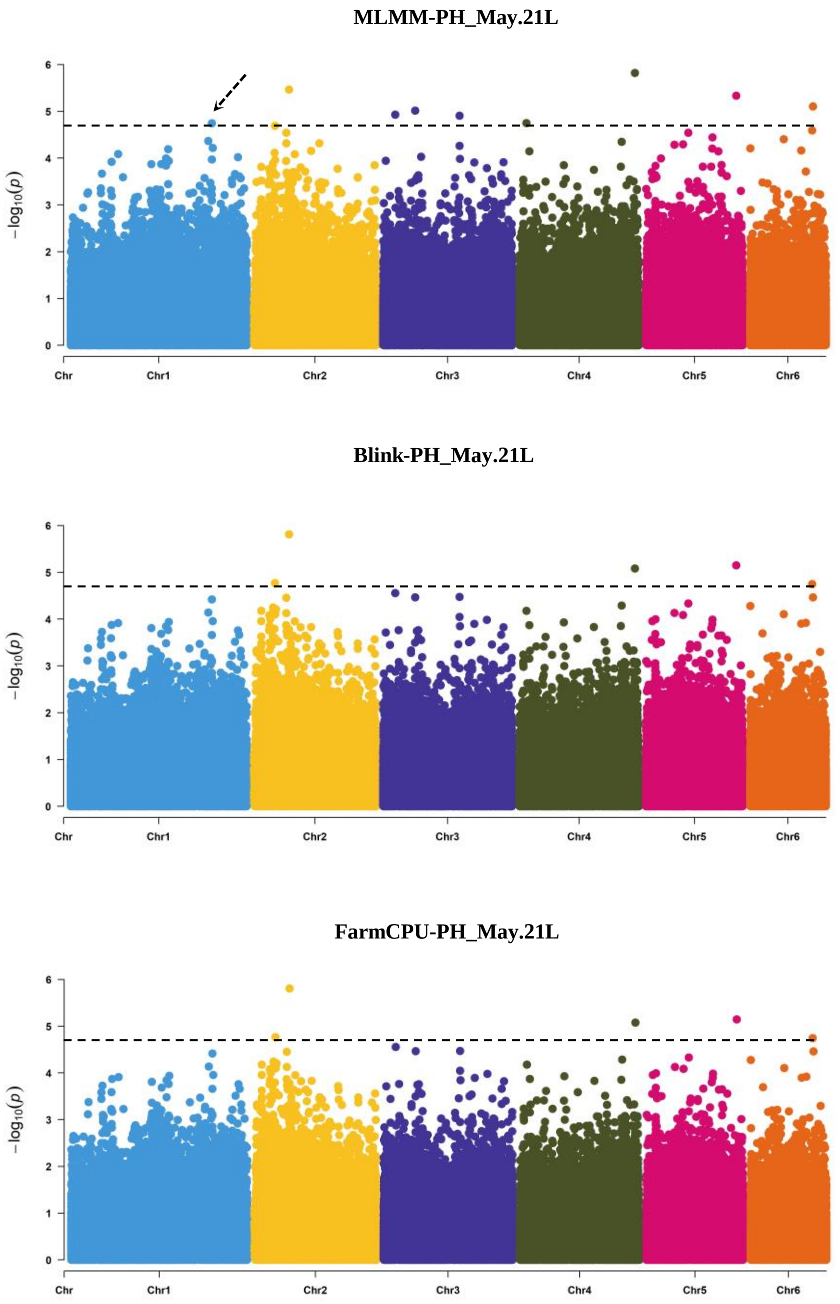

Fig. S18 Manhattan plots for plant height of 241 accessions in May 2021 LiangShan using MLMM, Blink and FarmCPU. The black dashed lines indicate the significance threshold (p value = 2.0×10^{-5}) and black arrow indicates the significant GWAS peak.
